# Mouse genome rewriting and tailoring of three important disease loci

**DOI:** 10.1038/s41586-023-06675-4

**Published:** 2023-11-01

**Authors:** Weimin Zhang, Ilona Golynker, Ran Brosh, Alvaro Fajardo, Yinan Zhu, Aleksandra M. Wudzinska, Raquel Ordoñez, André M. Ribeiro-dos-Santos, Lucia Carrau, Payal Damani-Yokota, Stephen T. Yeung, Camille Khairallah, Antonio Vela Gartner, Noor Chalhoub, Emily Huang, Hannah J. Ashe, Kamal M. Khanna, Matthew T. Maurano, Sang Yong Kim, Benjamin R. tenOever, Jef D. Boeke

**Affiliations:** 1 Institute for Systems Genetics and Department of Biochemistry and Molecular Pharmacology, NYU Langone Health, New York, NY USA; 2 Department of Microbiology, NYU Langone Health, New York, NY USA; 3 Perlmutter Cancer Center, NYU Langone Health, New York, NY USA; 4 Department of Pathology, NYU Langone Health, New York, NY USA; 5 Department of Medicine, NYU Langone Health, New York, NY USA; 6 Department of Biomedical Engineering, NYU Tandon School of Engineering, Brooklyn, NY USA

**Keywords:** Synthetic biology, Genetics

## Abstract

Genetically engineered mouse models (GEMMs) help us to understand human pathologies and develop new therapies, yet faithfully recapitulating human diseases in mice is challenging. Advances in genomics have highlighted the importance of non-coding regulatory genome sequences, which control spatiotemporal gene expression patterns and splicing in many human diseases^1,2^. Including regulatory extensive genomic regions, which requires large-scale genome engineering, should enhance the quality of disease modelling. Existing methods set limits on the size and efficiency of DNA delivery, hampering the routine creation of highly informative models that we call genomically rewritten and tailored GEMMs (GREAT-GEMMs). Here we describe ‘mammalian switching antibiotic resistance markers progressively for integration’ (mSwAP-In), a method for efficient genome rewriting in mouse embryonic stem cells. We demonstrate the use of mSwAP-In for iterative genome rewriting of up to 115 kb of a tailored *Trp53* locus, as well as for humanization of mice using 116 kb and 180 kb human *ACE2* loci. The *ACE2* model recapitulated human *ACE2* expression patterns and splicing, and notably, presented milder symptoms when challenged with SARS-CoV-2 compared with the existing *K18-hACE2* model, thus representing a more human-like model of infection. Finally, we demonstrated serial genome writing by humanizing mouse *Tmprss2* biallelically in the *ACE2* GREAT-GEMM, highlighting the versatility of mSwAP-In in genome writing.

## Abstract (web summary)

This study describes a method to insert large stretches of exogenous DNA into mammalian genomes, which is used to insert human *ACE2* loci into mouse to produce a model of human SARS-CoV-2 infection.

## Main

Genome synthesis is feasible for prokaryotes such as *Escherichia coli*^3^, *Mycoplasma*^4^ and eukaryotes such as *Saccharomyces cerevisiae*^5–10^. However, mammalian genome synthesis remains prohibitive owing to genome size and complexity^11^. An intermediate step is to overwrite large swaths of a native genomic region that covers a full locus, complete with all regulatory regions and/or several nearby genes. The combination of large DNA (over 100 kb) assembly approaches with the use of site-specific recombinases in mammalian systems has proved to be an efficient method for large-scale modification of mammalian genomes^12,13^. Previous delivery methods for large DNA fragments were limited by scars left behind in the genome^14^, a problem largely solved by the recently developed Big-IN method^15^; however, current methods are not usually designed for iterative deliveries, limiting the total size of the delivered DNA. A cleaner, more efficient mammalian genome writing method that can, in theory, be used to overwrite entire mammalian chromosomes will be broadly useful.

Mouse embryonic stem (ES) cells are relatively easy to genetically manipulate, and the subsequent derivation of mouse models is enabled by the generation of chimeras or tetraploid complementation^16^. Genetically humanizing mouse loci can bridge human–mouse evolutionary gaps, which are reflected in some cases by the lack of clear human orthologues in mice^17^ and the inability to recapitulate human disease^18,19^. Transgenesis—in which a human coding sequence is controlled by a strong heterologous promoter—is the predominant approach for mouse humanization, and results in non-physiological expression patterns. Projects such as Encyclopedia of DNA Elements^1^ (ENCODE) and genome-wide association studies^2^ (GWAS) established the importance of non-coding regulatory elements, making full genomic humanization (including non-coding regions) preferable. Human bacterial artificial chromosome (BAC)-based transgenes retain full-length human gene sequences, but are often randomly integrated^20^, leading to idiosyncratic position effects, not reliably mimicking the endogenous genomic context and thus compromising authentic expression patterns. Precision tailoring of BACs and in situ rewriting of their mouse counterpart(s) represent enhanced strategies for addressing these shortcomings, with previous work on in situ humanization of mouse immunoglobulin genes as an example. However, the overall efficiency for each human sequence integration in those methods was low^21,22^, limiting their widespread adoption.

During the COVID-19 pandemic, one of many substantial challenges was the limitations of mouse models for understanding human disease physiology. Owing to coding polymorphisms in the mouse version of the viral receptor ACE2, original isolates such as the Washington strain are unable to productively infect mice. Although the virus can be adapted to mice^23^, studying the biology of a modified virus limits the value of the model. Similarly, current animal models in which human *ACE2* is genetically introduced as a transgene^19^ can lead to changes in viral tropism that are not observed physiologically. Although recent variants of SARS-CoV-2 have gained some capacity to infect mice^24^, the host response does not phenocopy the human disease course. Therefore, a mouse model that is susceptible to SARS-CoV-2 and better mimics human pathology could be valuable for therapeutic development and lead to improved basic understanding of the effects of age, immune suppression and other factors on viral disease. Further, such models could leverage vast mouse genetic resources and might help prepare against future disease outbreaks. Transgenic *ACE2* mouse models developed in response to severe acute respiratory syndrome coronavirus (SARS-CoV) and Middle East respiratory syndrome coronavirus (MERS-CoV) outbreaks provided good platforms for understanding these diseases^19,25^. Yet, they have limitations: (1) they lack the human regulatory elements around *ACE2* and cannot recapitulate the spatiotemporal regulation of human *ACE2*; (2) mouse *Ace2* may lack splicing signals that are required to produce certain human-specific isoforms^26^; and (3) transgenic mice have an intact endogenous *Ace2*, resulting in convoluted expression of both human and mouse receptors. A genomically humanized *ACE2* mouse that more accurately models coronavirus diseases is urgently needed.

Here we report mSwAP-In, a novel mammalian genome writing method for large-scale efficient, scarless, iterative and biallelic genome writing in mouse ES cells. Before the COVID-19 pandemic, we developed mSwAP-In to address the challenge set by Genome Project-Write^27^: to engineer a synthetic *Trp53* tailored with recoded mutational hotspots that are predicted to render cells more resistant to spontaneous oncogenic *Trp53* mutations. This platform highlights the utility of mSwAP-In for the delivery of synthetic mouse genes, and for iterative genome writing using three carefully designed secondary *Trp53* downstream payload DNAs. To build an improved mouse model of COVID-19, we swapped 72 kb of mouse *Ace2* with 116 kb or 180 kb of human *ACE2*. The subsequently-generated *ACE2* GREAT-GEMM accurately reflected human-specific transcription and splicing patterns. *ACE2* humanized mice were susceptible to SARS-CoV-2 upon intranasal infection, but unlike the *K18-hACE2* transgenic mouse, these mice did not succumb to infection, suggesting that *ACE2* GREAT-GEMMs are a better model for COVID-19 in humans. Finally, we demonstrated the biallelic genome writing capability of mSwAP-In by overwriting mouse *Tmprss2* with human *TMPRSS2* in *ACE2* humanized mouse ES cells, resulting in double-humanized *ACE2* and *TMPRSS2* mice.

## Design of mSwAP-In

**Fig. 1:**
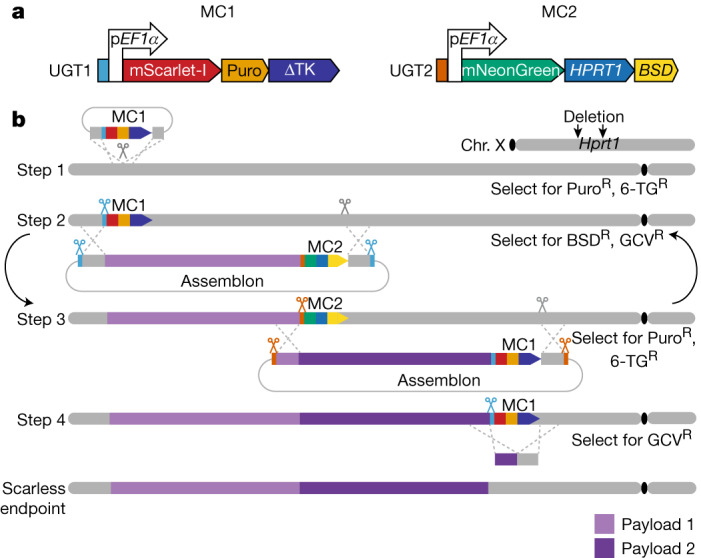
The mSwAP-In strategy for genome writing. **a**, Two interchangeable marker cassettes (MC1 and MC2) underlie mSwAP-In selection and counterselection. BSD, blasticidin S deaminase; Puro, puromycin resistance gene; ΔTK, truncated version of HSV1 thymidine kinase. **b**, Stepwise genome rewriting using mSwAP-In. A prior engineering step to delete endogenous *Hprt1* enables later iteration. Step 1: integration of MC1 upstream of locus of interest. Step 2: delivery of payload DNA including MC2 and Cas9–gRNAs for integration through HR. Step 3: delivery of next payload DNA following the same strategy as step 2, swapping back to MC1. Iterative steps 2 and 3 can be repeated indefinitely using a series of synthetic payloads by alternating selection for MC1 and MC2 (curved arrows). Step 4: removal of final MC1 or MC2. Grey bars are native chromosome regions; purple bars are synthetic incoming DNAs; blue and brown scissors are universal Cas9–gRNAs that cut UGT1 and UGT2, respectively; grey scissors are genome-targeting Cas9–gRNAs. Superscript R indicates resistance to puromycin (Puro^R^), 6-thioguanine (6-TG^R^), blasticidin (BSD^R^) or ganciclovir (GCV^R^). Chr., chromosome.

**Extended Data Fig. 1:**
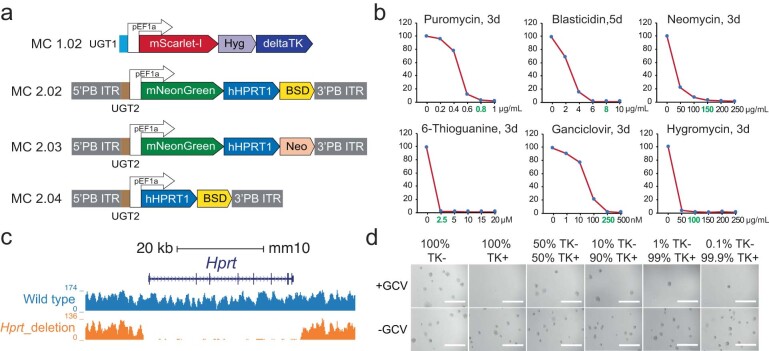
mSwAP-In design and development. (**a**) Alternative marker cassettes compatible with genetic backgrounds harboring preexisting drug resistance genes. PB ITR, piggyBac inverted terminal repeat; UGT, universal gRNA target. (**b**) mESC kill curve for each mSwAP-In selection marker. Selected concentrations are highlighted in green: 0.8 μg/ml for puromycin, 8 μg /ml for blasticidin, 150 μg/ml for neomycin, 2.5 μM for 6-thioguanine, 250 nM for ganciclovir and 100 μg/ml for hygromycin. (**c**) Capture-seq analysis of *Hprt* deletion. Sequencing reads were mapped to mm10. (**d**) The bystander effect of thymidine kinase can be overcome by plating single colonies. As few as 0.1% TK-negative cells can be isolated. GCV, ganciclovir (250 nM). Scale bar is 1 mm.

Most genome engineering methods are restricted by difficulties in DNA assembly, purification and delivery to mammalian cells as construct length increases. To overcome size limitations, we developed mSwAP-In, a method descended from the yeast genome rewriting method, SwAP-In^5,7^. Two types of marker cassettes (MC1 and MC2) were designed (Fig. 1a), each with a distinct set comprising: (1) a fluorescence marker indicating correct DNA swaps; (2) a positive selection marker; and (3) a negative selection marker overwritten with mouse DNA in each swapping step, that selects against off-target integrations. A series of marker cassettes was designed to accommodate genetic backgrounds already containing selectable markers (Extended Data Fig. 1a) and tested for effective elimination of sensitive mouse ES cells (Extended Data Fig. 1b). A universal guide RNA (gRNA) target (UGT) site orthogonal to mammalian genomes (derived from GFP) was placed in front of each marker cassette to enable specific and efficient cleavage by Cas9 or other nucleases. To deploy the *HPRT1* minigene in MC2 in later mSwAP-In stages, mouse ES cells were pre-engineered to delete endogenous *Hprt1* using two Cas9–gRNAs followed by 6-thioguanine selection (Extended Data Fig. 1c). mSwAP-In is executed in several steps: (1) MC1 is inserted at a ‘safe’ location near the genomic region of interest using CRISPR–Cas9 assisted homologous recombination (HR) (Fig. 1b, step 1). (2) A synthetic payload DNA (called an assemblon^28^), consisting of flanking UGT1 sites, homology arms (approximately 2 kb at each end) and MC2, is pre-assembled in yeast and then co-delivered with two Cas9–gRNAs that recognize UGT1 and the distal native genomic segment boundary to be overwritten (Fig. 1b, step 2). Integration by HR is promoted by payload DNA linearization at two flanking UGT1 sites and by double strand breaks at the target. Successful targeted cells are selected using the positive selection marker of MC2 (blasticidin S deaminase) and the negative selection marker of the parental MC1 (a truncated version of herpes simplex virus 1 thymidine kinase) (Extended Data Fig. 1d), resulting in overwriting of the wild-type segment by synthetic payload DNA. This process is iterated in step 3 with a second synthetic payload DNA, assembled similarly in yeast with homology arms and MC1, by positive selection using the puromycin resistance gene in MC1 and negative selection against the *HPRT1* in MC2 (Fig. 1b, step 3). The iteration can in principle continue indefinitely as needed. Once overwriting is complete, there is the option to remove the last marker cassette using CRISPR–Cas9-assisted HR or PiggyBAC excision^29^, producing scarless engineered cells (Fig. 1b, step 4).

## Rewriting the *Trp53* locus with mSwAP-In

**Fig. 2:**
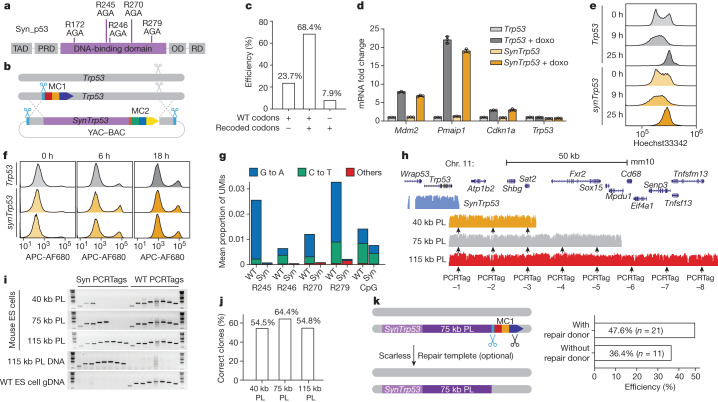
Rewriting the *Trp53* locus with mSwAP-In. **a**, Design of p53 hotspot mutation recoding. Top, recoded codons. OD, oligomerization domain; PRD, proline-rich domain; RD, regulatory domain; TAD, transactivation domain. **b**,**c**, Schematic (**b**) and efficiency (**c**) of *synTrp53* mSwAP-In. WT, wild type. **d**, Functional evaluation of recoded *synTrp53*. Mouse ES cells with either WT *Trp53* or *synTrp53* were treated with 250 nM of doxorubicin (doxo) for 20 h. Levels of *Mdm2*, *Pmaip1*, *Cdkn1a* and *Trp53* mRNA were evaluated by RT–qPCR. mRNA levels were normalized to *Actb*. Data are mean ± s.d. of three technical replicates. **e**, Histogram of DNA content in mouse ES cells stained with Hoechst33342. **f**, Histogram of Alexa Fluor 680 conjugated to annexin V, the showing apoptotic cell population. **g**, Mutation frequencies at four mutational hotspots and remaining (non-recoded) CpG sites in *Trp53* WT or *synTrp53* (syn) mouse ES cells. Mutation frequencies were calculated by averaging UMI frequencies of analysed codons or dinucleotides. R155 and R172 codons from first amplicon were excluded because a similar genomic sequence was amplified from a *Trp53* pseudogene, rendering those data uninformative. **h**, Sequence coverage of *synTrp53* and three *Trp53* downstream tailored payloads (PL) aligned to mm10. Arrows indicate positions of PCRTags. **i**, Genotyping of three representative mSwAP-In integrants from three *Trp53* downstream PL. **j**, Summary of mSwAP-In success rates based on genotyping. **k**, Strategy for final marker cassette removal and genotyping-based summary of efficiency. Blue scissors indicate UGT1-targeting gRNA; black scissors indicate gRNA targeting the SV40 terminator.

**Extended Data Fig. 2:**
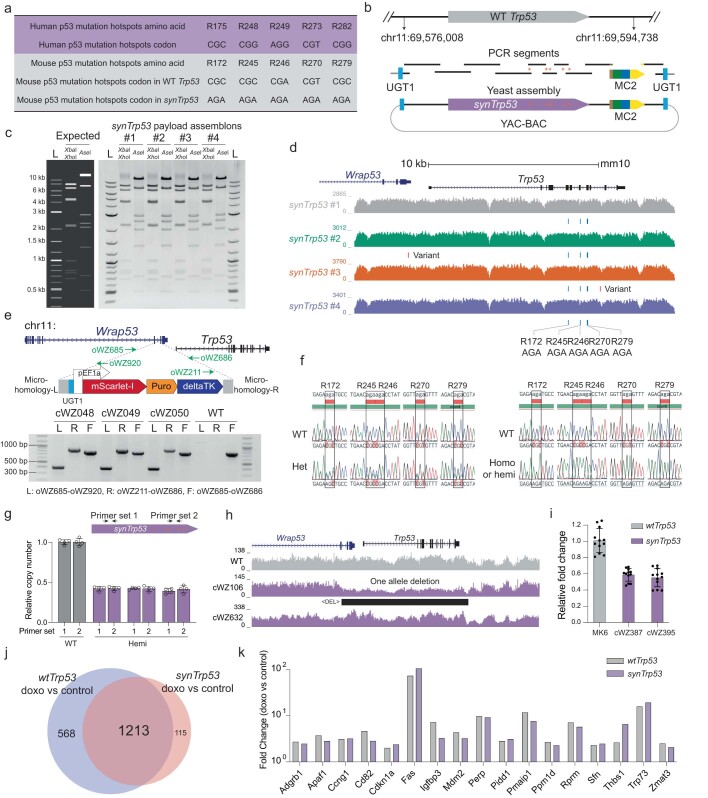
Synthetic *Trp53* integration via mSwAP-In. (**a**) p53 mutation hotspots and the corresponding DNA codons in human and mouse, as well as the recoded codons in *synTrp53*. (**b**) *SynTrp53* assembly workflow. Red asterisks represent the recoded codons. (**c**) Restriction enzyme digestion verification of *synTrp53* assemblons. *Xba*I+*Xho*I and *Ase*I were used for each candidate. Predicted digestion patterns were simulated using Snapgene software. L, 1 kb plus ladder (NEB). (**d**) Sequencing coverage of *synTrp53* payload candidates. Reads were mapped to mm10 reference. Clones 1 and 3 have expected variants reflecting the recoded codons in synthetic *Trp53*. Clone 2 and clone 4 have additional undesired variants likely introduced by PCR. (**e**) Marker cassette 1 insertion into the second intron of *Wrap53*. 20 bp microhomology arms were added to each end of MC1 during plasmid construction. Successful insertion was verified by junction PCR. Primers are indicated as green arrows. (**f**) Sanger sequencing validation of heterozygous and hemizygous *synTrp53* integrants. (**g**) *Trp53* copy number qPCR analysis for the three mESC clones only carrying recoded codons. *Trp53* copy number was normalized to *Pgk1* gene. Bars represent mean ± SD of three technical replicates. (**h**) Sequencing coverage for wild-type, hemizygous and heterozygous clones. Sequencing reads were mapped to mm10. Black bar indicates a deletion called by DELLY^50^. (**i**) RT-qPCR analysis of *Trp53* expression level. Bars represent mean ± SD of four technical replicates for cell culture and RNA extraction, as well as three qPCR technical replicates for each reverse transcribed cDNA sample. (**j**) Venn diagram of upregulated gene numbers in *Trp53*^*wt/wt*^ and *Trp53*^*syn/syn*^ mESCs upon doxorubicin (250 nM for 20 h) treatment. Fold change cutoff is 4, adjusted p value cut off is 0.01. (**k**) A fold change representation of 17 genes in the p53 signaling pathway in doxorubicin treated (250 nM for 20 h) *Trp53*^*wt/wt*^ and *Trp53*^*syn/syn*^ mESCs.

We sought to engineer a ‘cancer-mutation-resistant’ *Trp53* (ref. ^27^) (the gene encoding p53) in mouse ES cells using mSwAP-In. Missense p53 mutations occur frequently in cancer and are concentrated at CG sites^30^ in the DNA binding domain, owing to frequent deamination of 5-methylcytosine leading to C to T conversion^31^ and binding of DNA adducts to certain methylated CGs^32^. We hypothesized that synonymously recoding *Trp53* DNA to avoid CG dinucleotides would minimize such mutations; we therefore recoded CGs in p53 mutation hotspots (R172, R245, R246, R270 and R279) to AG (Fig. 2a and Extended Data Fig. 2a).

Synthetic recoded *Trp53* (*synTrp53*) was assembled in a yeast assembly vector (YAV) (Extended Data Fig. 2b) and verified by sequencing and restriction digestion (Extended Data Fig. 2c,d). In parallel, we inserted MC1 downstream of mouse *Trp53* heterozygously (Extended Data Fig. 2e). After deploying mSwAP-In with the *synTrp53* payload into MC1-containing mouse ES cells, 87.1% of colonies lost MC1 and gained MC2 (*n* = 132). We analysed 38 genotype-verified clones by Sanger sequencing and found that 26 of these clones carried the recoded codons in one of the two alleles, 9 were unedited, and 3 of them only carried the recoded *synTrp53* (Fig. 2b,c and Extended Data Fig. 2f). *Trp53* copy number analysis of those three clones carrying only recoded codons suggested that they were hemizygous (Extended Data Fig. 2g); this was confirmed by Capture-seq^15^ (Extended Data Fig. 2h). To ensure that mSwAP-In engineering was free of off-target effects, we implemented bamintersect analysis^15^, a modular mapping tool that detects reads spanning two references (for example, payload DNA versus mm10, or homology arm versus mm10). This analysis detected no off-target junctions in the six sequenced clones; YAV backbone integration was seen in one clone (Supplementary File 1). *SynTrp53* heterozygotes can be further engineered to homozygotes by repeating mSwAP-In on the wild-type allele, but using a different version of MC2. To test *synTrp53* function, we treated *Trp53* wild-type and homozygous *synTrp53* mouse ES cell lines with doxorubicin. Three classical p53 target genes—*Mdm2*, *Pmaip1* (which encodes Noxa) and *Cdkn1a* (which encodes p21)—were upregulated in *synTrp53* mouse ES cells to a similar degree as in *Trp53* wild-type mouse ES cells (Fig. 2d). Of note, with only six CpG sites removed from the *synTrp53* gene body, the *Trp53* expression level was 30–40% lower in the *synTrp53* mouse ES cells (Fig. 2d and Extended Data Fig. 2i), consistent with previous observations suggesting that DNA methylation in the gene body is associated with higher gene expression^33,34^. Transcript profiling of doxorubicin-treated *Trp53* wild-type and *synTrp53* mouse ES cells revealed similar global stress responses (Extended Data Fig. 2j,k), thus *Trp53* recoding did not impair its transactivation function. Additionally, both wild-type and *synTrp53* ES cells underwent growth arrest (Fig. 2e and Supplementary Fig. 1) and cell apoptosis in response to doxorubicin treatment (Fig. 2f and Supplementary Fig. 2).

**Extended Data Fig. 3:**
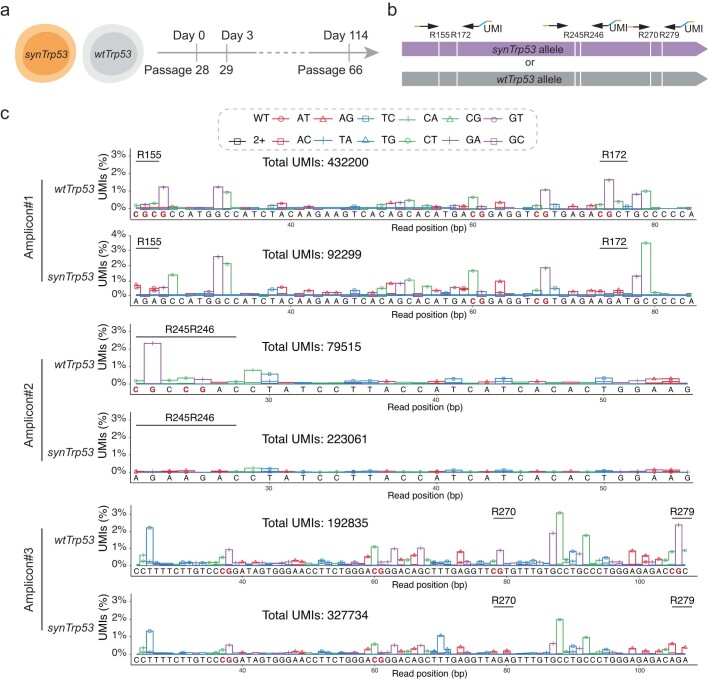
Spontaneous mutation frequencies in *wtTrp53* and *synTrp53* mESCs. (**a**) Experimental design. *Trp53*^*syn/syn*^ and *Trp53*^*wt/wt*^ clones were passaged every three days for a total of 38 passages, to accumulate spontaneous mutations. (**b**) Amplicon-seq design. The six recoded codons in *Trp53* gene were amplified in three amplicons. Black arrows are primer annealing sequences, blue lines are unique molecular identifiers (UMIs), orange lines are Illumina indexes. (**c**) UMI frequencies for all sequenced base pairs in *wtTrp53* and *synTrp53* mESCs.

To investigate whether recoding mutation hotspots of *Trp53* makes *synTrp53* more resistant to spontaneous mutagenesis, we grew *Trp53* wild-type and *synTrp53* mouse ES cells for a total of 38 passages to enable mutation accumulation. We used a unique molecular identifier (UMI)-based amplicon sequencing method^35^ to measure hotspot mutation frequencies (Extended Data Fig. 3a,b). C>T and G>A mutations were observed at high frequency in wild-type *Trp53* hotspot codons, but not in *synTrp53* hotspot codons, and recoded AGA codons had much lower mutation frequencies (Fig. 2g); no significant differences were seen comparing other codons between samples (Extended Data Fig. 3c).

**Extended Data Fig. 4:**
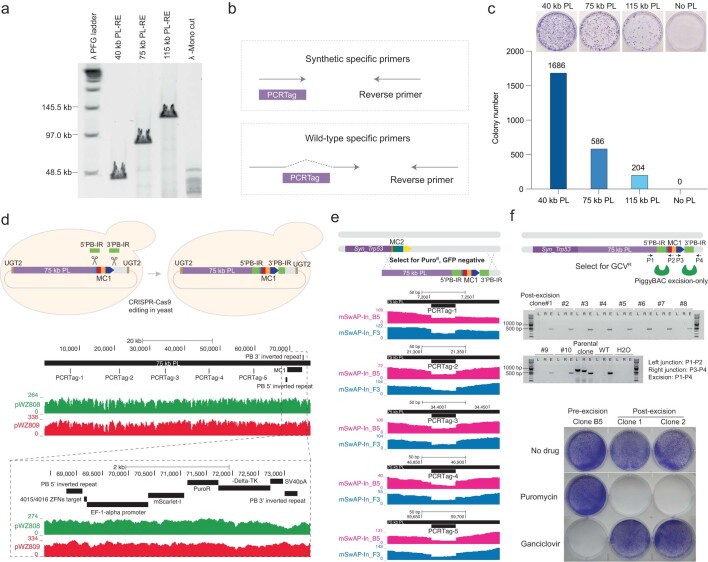
Iterative mSwAP-In and marker cassette removal. (**a**) Pulse field gel electrophoresis analysis of three *Trp53* downstream payloads linearized with a single-cutter. PL-RE, payload DNA digested with restriction enzyme. (**b**) Synthetic and wild-type specific PCR assays employing a specific forward primer and a universal reverse primer. (**c**) Total colony number for the 40 kb, 75 kb and 115 kb payload deliveries using mSwAP-In. mESC colonies were fixed and stained with crystal violet. (**d**) CRISPR-Cas9 was used in yeast to facilitate insertion of the 5’ and 3’ piggyBAC inverted terminal repeat sequences into the flanking sites of marker cassette 1 within the 75 kb payload. (**e**) Engineered 75 kb payload containing piggyBAC adaptors from panel (d) was integrated into *synTrp53* mESCs via mSwAP-In, and validated via capture-sequencing. (**f**) PiggyBAC excision-only transposase was employed to excise marker cassette 1 under negative (ganciclovir) selection. Ten clones were randomly chosen and genotyped by PCR, with two of the ten clones further tested for resistance to puromycin and ganciclovir.

To demonstrate iterability of mSwAP-In and to probe the upper genome writing length limit of each mSwAP-In step, we built 40-kb, 75-kb and 115-kb payload constructs using *Trp53* downstream DNA for a second round of mSwAP-In (Fig. 2h and Extended Data Fig. 4a). To distinguish synthetic and native DNA in subsequent steps, watermarks were tailored in approximately every 13 kb of sequence in intronic or intergenic regions; these ‘PCRTag’ watermarks which are 28-bp orthogonal DNA sequences (Supplementary Table 1) that resemble the PCRTags used in Sc2.0 (ref. ^7^). Synthetic- or native-specific primer pairs distinguished the sequences (Extended Data Fig. 4b). After deploying mSwAP-In into a heterozygous *synTrp53* mouse ES cell clone, we observed a gain of synthetic PCRTags for delivered payloads as well as the native PCRTags, indicating heterozygous integration (Fig. 2i). Although the total drug-resistant colony number decreased inversely with payload length (Extended Data Fig. 4c), the efficiency of mSwAP-In remained above 50% (Fig. 2j).

Finally, we demonstrated the feasibility of marker cassette removal; the efficiency of MC1 removal was 47.6% when providing a repair template of around 2 kb and CRISPR–Cas9 reagents, and 36.4% when no repair template was provided (Fig. 2k); when using piggyBAC, 100% of clones lost MC1 (Extended Data Fig. 4d–f). Collectively, these data show that mSwAP-In is efficient for large-scale iterative and scarless genome rewriting in mouse ES cells. However, all payloads that we delivered up to this point were more than 99% identical to the native mouse sequences, which might have contributed to the high efficiency. Next, we tested whether mSwAP-In could overwrite the native genome with nonhomologous DNA, such as entire human loci.

## Fully humanizing *ACE2* in mouse ES cells

**Fig. 3:**
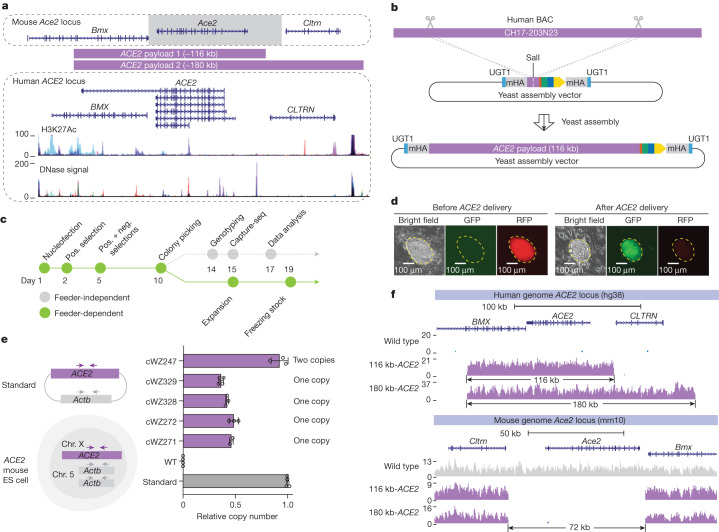
Fully humanizing *ACE2* in mouse ES cells. **a**, Genome browser screenshots of mouse *Ace2* and human *ACE2* loci. H3K27 acetylation and DNase signal tracks in the *ACE2* locus indicate functional regulatory elements. The grey box demarcates the overwritten mouse genomic region. Purple bars demarcate human genomic regions included in *ACE2* payloads. **b**, *ACE2* payload assembly strategy. Scissors mark in vitro CRISPR–Cas9 digestion sites. mHA, mouse homology arm. **c**, Mouse ES cell engineering workflow. Neg., negative; pos., positive. **d**, Representative images of fluorescence marker switching in outlined mouse ES cell clones. More than 80% of mouse ES cell clones exhibited the expected fluorescence switch; the mSwAP-In experiment was repeated at least three times with similar results. **e**, *ACE2* copy number determination by qPCR. The ratio between *ACE2* and *Actb* is 0.5, indicating that a single copy of *ACE2* was delivered to male mouse ES cells, as expected. Copy number was normalized to *Actb*. Data are mean ± s.d. of three technical replicates. **f**, Sequencing coverage of 116 kb-*ACE2* and 180 kb-*ACE2* mSwAP-In clones. Reads were mapped to hg38 (top) and mm10 (bottom).

**Extended Data Fig. 5:**
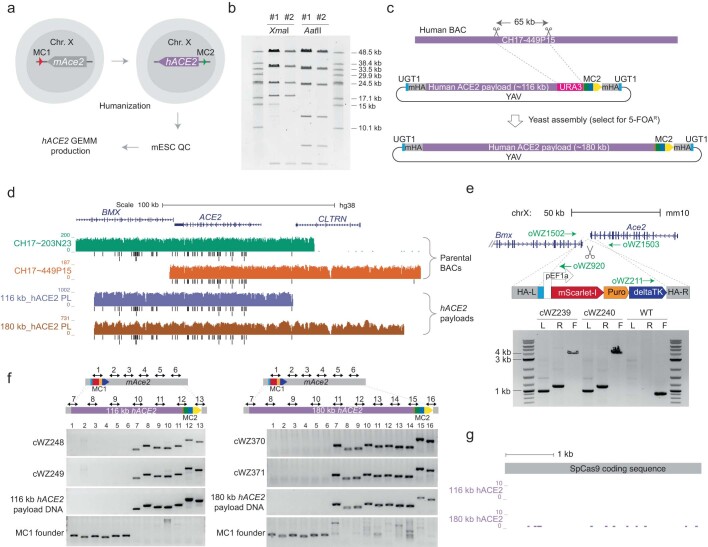
Genomically rewriting mouse *Ace2* with human *ACE2*. (**a**) Schematic workflow. (**b**) Restriction enzyme digestion verification of the *116 kb-hACE2* payload. Digestion products were separated using low-melting-point agarose gel by pulse field gel electrophoresis (see methods). (**c**) *180 kb-h*ACE2 payload assembly. Scissors mark in vitro CRISPR-Cas9 digestion sites. mHA, mouse homology arm. YAV, yeast assembly vector. (**d**) Sequencing coverage of two human BACs and the two *hACE2* payloads mapped to hg38. Black bars represent single nucleotide polymorphisms (SNPs). (**e**) Marker cassette 1 insertion to the downstream of mouse *Ace2*. Integration was confirmed by junction PCR. HA-L, left homology arm, HA-R, right homology arm. L, left junction assay with primers oWZ1502 and oWZ920. R, right junction assay with primers oWZ211 and oWZ1503. F, full MC1 amplification with primers oWZ1502 and oWZ1503. (**f**) Genotyping PCR analysis of *116 kb- hACE2* and *180 kb-hACE2* mSwAP-In clones. Double headed arrows mark PCR amplicons from either m*Ace2* (assays 1–6) or *hACE2* payloads (assays 7–16). (**g**) Sequencing coverage of Cas9 in *116 kb- hACE2* and *180 kb-hACE2* mESCs.

Mice are naturally resistant to SARS-CoV-2 owing to key residues in ACE2 that bind the viral spike protein^36^. However, the *K18-hACE2* transgenic mouse—in which a keratin 18 promoter drives high expression of human *ACE2* mRNA in epithelial tissues, including respiratory epithelia—is readily infected^19^, resulting in 100% of infected mice dying in days^37^, a phenotype that is not observed in humans. To establish a more physiological model, we aimed to use mSwAP-In to completely swap the mouse *Ace2* locus with the human *ACE2* locus, including all introns and regulatory elements (Fig. 3a and Extended Data Fig. 5a). On the basis of gene annotation, we found a long transcript (NM_001386259.1, also known as transcript variant 3) that spans 83 kb and largely overlaps with the *BMX* gene (Fig. 3a). In contrast to the canonical transcript that encodes an 805-amino-acid protein, the long transcript encodes a 786-amino-acid ACE2 protein lacking an intact collectrin homology domain at the C terminus and instead including a novel 16-amino-acid exon^38^. To maximize retention of function, we defined a left payload boundary to include the long transcript. For the right boundary, considering DNase-hypersensitive sites and H3K27 acetylation marks, we designed two *ACE2* payloads: one extending to the 3′ end of *CLTRN* (116 kb-*ACE2*), and one extending beyond the 5′ end of *CLTRN* (180 kb-*ACE2*) (Fig. 3a).

The 116 kb-*ACE2* region from human BAC CH17-203N23 was assembled through yeast HR into an acceptor vector^15^ containing flanking UGT1 sites, left and right *Ace2* homology arms and MC2 (Fig. 3b), and verified by restriction digestion (Extended Data Fig. 5b). The 180 kb-*ACE2* payload was built by inserting an additional 64-kb fragment released from BAC CH17-449P15 into the end of the 116 kb-*ACE2* payload (Extended Data Fig. 5c). Sequencing revealed no variants within the two payloads, except single nucleotide polymorphisms (SNPs) present in parental BACs, highlighting the high accuracy of this HR- and BAC-based assembly workflow (Extended Data Fig. 5d). To enable payload delivery using mSwAP-In, we inserted MC1 downstream of *Ace2* in mouse ES cells (Extended Data Fig. 5e). We used feeder-dependent cell culture conditions to maintain the developmental potential of the mouse ES cells, splitting cells into feeder-independent subcultures for structural analysis (Fig. 3c). We delivered both *ACE2* payloads into MC1 founder line with mSwAP-In, observing the expected fluorescent marker switch (Fig. 3d). To ensure that *Ace2* locus was fully overwritten, we performed genotyping PCR using multiple primers across *Ace2* and *ACE2* regions. Correct clones showed presence of *ACE2* amplicons and absence of *Ace2* amplicons (Extended Data Fig. 5f). The overall efficiency was 61.5% (*n* = 13) for the 116 kb-*ACE2* payload, and 60.8% (*n* = 79) for the 180 kb-*ACE2* payload, as determined by genotyping.

To enable *ACE2* copy number quantification, we constructed a plasmid containing one copy of mouse *Actb* and one copy of human *ACE2* to serve as an internal standard for quantitative PCR (qPCR), and identified mouse ES cell clones with one copy of *ACE2* (Fig. 3e); Capture-seq verified that the *ACE2* clones lacked deletions or duplications, as well as the loss of mouse *Ace2* (Fig. 3f). No off-target integration was revealed by bamintersect analysis (Supplementary File 1), and no Cas9 or vector reads were captured in these mouse ES cell clones (Extended Data Fig. 5g). Considering all steps of this comprehensive sequence quality control, the overall success rates for the delivery of 116 kb-*ACE2* and 180 kb-*ACE2* payloads were 15.4% and 22.8%, respectively.

## *ACE2* expression and epigenetic landscape

**Fig. 4:**
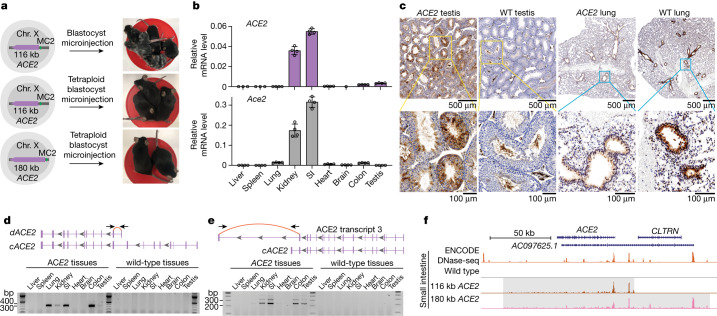
Characterization of *ACE2* expression in mouse. **a**, Production of *ACE2* mice via injection of chimeric blastocyst and tetraploid blastocyst embryos. **b**, RT–qPCR analysis of *ACE2* (top) and *Ace2* (bottom) expression in nine tissues collected from four-week-old *ACE2* and wild-type mice. Expression was normalized to mouse *Actb*. Data are mean ± s.d. of three technical replicates. SI, small intestine. **c**, Immunohistochemistry analysis of ACE2 in testis and lung dissected from ten-week-old *ACE2* or wild-type mice. The antibody reacts with both human and mouse ACE2. Yellow and blue boxes mark magnified areas. *n* = 2 independent mice for each tissue; the immunohistochemistry experiment was repeated twice. **d**, PCR with reverse transcription (RT–PCR) detection of *dACE2* isoform (transcript variant 5) in tissues from *ACE2* mice. *cACE2*, canonical *ACE2* transcript. Independent PCR assays were performed at least twice. **e**, Detection of *ACE2* transcript 3 in tissues from *ACE2* mice. **f**, ATAC–seq analysis of *ACE2* in *Ace2* wild-type, 116 kb-*ACE2* and 180 kb-*ACE2* small intestinal cells. A human small intestine DNase-seq track (ENCODE, DS20770) is displayed as a positive control. Shaded areas indicate *ACE2* regions.

**Extended Data Fig. 6:**
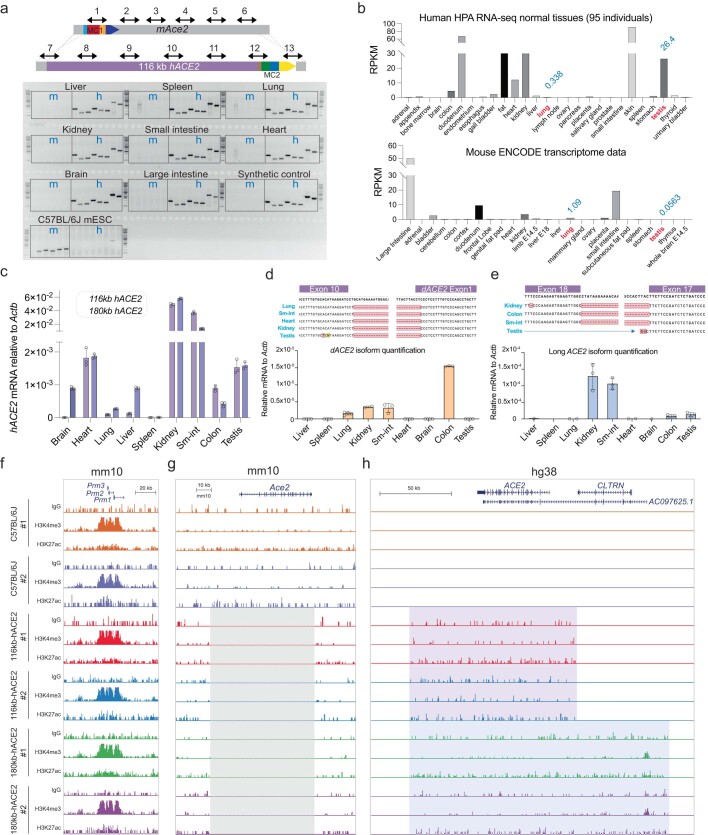
*hACE2* expression, slicing and epigenetic landscape characterization. (**a**) Genotyping PCR analysis of eight tissues from a tetraploid complementation-derived male. Double headed arrows are PCR amplicons from either mouse *Ace2* locus or human *ACE2* locus. m, *mAce2* amplicons; h, *hACE2* amplicons. (**b**) Human *ACE2* and mouse *Ace2* transcriptomic data from NCBI database. Lung and testis are highlighted in red with RPKM values indicated above. (**c**) *ACE2* expression profiling in *116kb-hACE2* and *180kb-hACE2* mouse models. RT-qPCR analysis of human *ACE2* in nine tissues of the two *ACE2* humanized mouse models. *ACE2* expression level was normalized to *Actb*. Bars represent mean ± SD of three technical replicates. (**d**-**e**) Two *hACE2* isoforms detected in *hACE2* mice. *dACE2* novel junction Sanger sequencing analysis (up) and tissue distribution (down) (d). *hACE2* transcript 3 junction Sanger sequencing analysis (up) and tissue (down) (e). Expression levels were normalized to *Actb* gene, bars represent mean ± SD of three technical replicates. (**f**-**h**) Genome browser shot of mouse testis specific *Prm1, Prm2, Prm3* locus (f), mouse *Ace2* locus (g) and human *ACE2* locus (h) loaded with CUT&RUN sequencing reads from IgG control, H3K4me3 and H3K27ac antibodies. Two biological replicates were used for each genotype. Greg box indicates the deleted mouse *Ace2* region, purple box indicates the 116 kb humanized *ACE2* region, light blue box indicates the 180 kb humanized *ACE2* region.

*ACE2* mouse ES cells that passed stringent verification were subjected to blastocyst embryo injection and tetraploid blastocyst embryo injection, which requires full developmental pluripotency. Pups exhibited a high rate of coat colour chimerism (31 out of 45 pups) when the mouse ES cells with the 116 kb-*ACE2* payload were injected into wild-type blastocysts (Fig. 4a). Several chimeric male mice showed 100% germline transmission. When injecting mouse ES cells with 116 kb-*ACE2* and 180 kb-*ACE2* payloads into a tetraploid blastocyst for embryo complementation, 14% (*n* = 50) and 22.9% (*n* = 70) birth rates were observed, respectively (Supplementary Table 2). We genotyped various tissues from a mouse derived by tetraploid complementation, and detected only *ACE2* amplicons, indicating that the mice were not chimeric (Extended Data Fig. 6a).

Proper spatial expression of *ACE2* is crucial for studying SARS-CoV-2 pathogenesis. We therefore examined expression patterns of *ACE2*. We first examined expression across 9 tissues from the 116 kb-*ACE2* GEMM (Fig. 4b). Abundant *ACE2* mRNA was detected in the small intestine and kidney, with moderate levels in testis and colon, indicating that the mouse machinery faithfully expressed *ACE2*. Overall, we observed similar expression patterns for *Ace2* and *ACE2* in mice, aside from a few important differences. For instance, we readily detected *ACE2* in testis, recapitulating the human expression pattern, but mouse *Ace2* is not expressed in testis (Fig. 4b and Extended Data Fig. 6b); thus *ACE2* mice may be useful as models of possible human testicular infection by SARS-CoV-2 (ref. ^39^). In addition, we observed lower *ACE2* expression in the lungs of *ACE2* mice compared with *Ace2* expression in wild-type mice, consistent with comparisons of human and mouse transcriptomes (Extended Data Fig. 6b). Next, we compared *ACE2* expression profiles between 116 kb-*ACE2* and 180 kb-*ACE2* models. *ACE2* expression was approximately 100-fold higher in brain, 3- to 5-fold higher in lung and liver, and 2- to 3-fold lower in small intestine and colon in the 180 kb-*ACE2* model (Extended Data Fig. 6c), indicating potential regulatory functions in the additional 64 kb of DNA in the 180 kb-*ACE2* model.

Immunohistochemistry of testes of the 116 kb-*ACE2* mice showed robust *ACE2* expression in Sertoli cells, spermatogonia and spermatocytes, reminiscent of *ACE2* expression in human testis^39^. By contrast, only a subset of spermatozoa cells expressed ACE2 protein in wild-type mouse testis (Fig. 4c and Supplementary Fig. 3). Immunohistochemistry of lungs showed *ACE2* expression in bronchioles of both *ACE2* and wild-type mice, with much lower levels observed in lungs from *ACE2* mice (Fig. 4c). These data suggest that the *ACE2* mice exhibit human tissue-specific gene expression patterns, including tissue-specific ACE2 expression that is present in humans but not in non-humanized animals.

Given that we swapped-in the entire *ACE2* gene, we examined whether human-specific splicing patterns would be recapitulated in the *ACE2* mice. A recent study identified *dACE2* as an interferon-stimulated *ACE2* isoform, although the product of this transcript is not a SARS-CoV-2 receptor. This hints at a potential role for alternative *ACE2* splicing^26^. We readily detected *dACE2* in the lung, kidney, small intestine and colon of *ACE2* mice (Fig. 4d and Extended Data Fig. 6d). In addition, the long transcript (variant 3; Fig. 3a) was detected in small intestine, kidney, brain and testis of *ACE2* mice (Fig. 4e and Extended Data Fig. 6e), further demonstrating recapitualtion of human physiological alternative splicing patterns of *ACE2* in *ACE2* mice.

We probed the epigenetic landscape of *ACE2* mice and compared it to human data. We used assay for transposase-accessible chromatin with sequencing (ATAC–seq) to assess chromatin accessibility in small intestinal cells, where *ACE2* expression is highest. Notably, samples from both 116 kb-*ACE2* and 180 kb-ACE2 samples displayed peaks that overlapped extensively with a DNase-seq dataset from ENCODE human small intestine tissue, demonstrating that human epigenome accessibility is well recapitulated in *ACE2* mice (Fig. 4f). We also performed CUT&RUN assays for H3K27ac and H3K4me3 histone modifications in testicular cells of *ACE2* mice. Testis-specific genes showed peaks indicating active chromatin (Extended Data Fig. 6f). However, no predominant peak was observed in wild-type testis *Ace2* or in humanized *ACE2* genomic regions, except for a H3K4me3 peak near the distal end of the 180 kb-*ACE2* region (Extended Data Fig. 6g,h). This result is consistent with existing ENCODE datasets, which show a lack of obvious H3K27ac and H3K4me3 peaks in the *ACE2* genomic region in human testicular cells.

## *ACE2* mice are susceptible to SARS-CoV-2

**Fig. 5:**
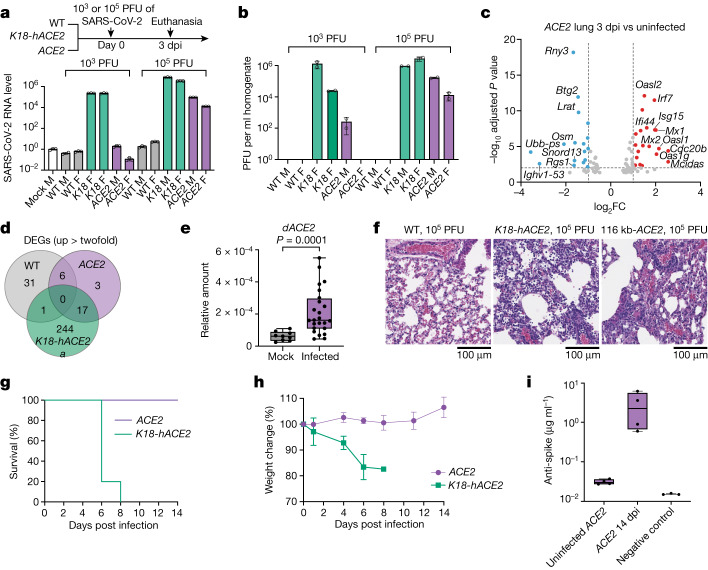
Characterizing the *ACE2* GEMM with SARS-CoV-2 infection. **a**,**b**, Lungs dissected from wild-type, *K18-hACE2* (*K18*) and 116 kb-*ACE2* (*ACE2*) mice infected with SARS-CoV-2 were analysed for nucleocapsid gene expression by RT–qPCR (**a**) and infectious viral levels by plaque assay (**b**). *n* = 4 independent mice for each group. SARS-CoV-2 levels were normalized to *Actb* and an uninfected control. F, female mice; M, male mice. **c**, Volcano plot of infected lungs versus uninfected lungs from 116 kb-*ACE2* mice. Red, upregulated genes in infected lungs; blue, downregulated genes in infected lungs. Fold change cut-off is set to 2; adjusted *P* value (Wald test) cut-off is set to 0.01. **d**, Venn diagram of upregulated (cut-off is twofold) differentially expressed genes (DEGs) in wild-type (WT), *K18-hACE2* and 116 kb-*ACE2* lungs. **e**, RT–qPCR analysis of *dACE2* in uninfected and SARS-CoV-2-infected lungs. *n* = 3 for uninfected lungs, *n* = 8 for infected lungs; 3 technical replicates were performed for each sample. Unpaired two-tailed, Mann–Whitney *t*-test. **f**, Histopathological analysis of lungs from female WT, *K18-hACE2* and 116 kb-*ACE2* mice by haematoxylin and eosin staining. Two lungs from independent infected mice were used; two spaced 5-μm sections from the same infected lung were stained and imaged. **g**,**h**, *K18-hACE2* (*n* = 5) and 116 kb-ACE2 (*n* = 4) mice were intranasally infected with 10^5^ PFU of SARS-CoV-2 and were monitored every other day for morbidity (**g**) and weight (**h**). Data are mean ± s.d. of biological replicates. **i**, Serological detection of anti-SARS-CoV-2 mouse IgG by ELISA. *n* = 4 independent mice for uninfected and infected groups. Box plots contain 25th to 75th percentiles of the data, the horizontal line in each box denotes the median value, and whiskers represent minima (low) and maxima (high).

**Extended Data Fig. 7:**
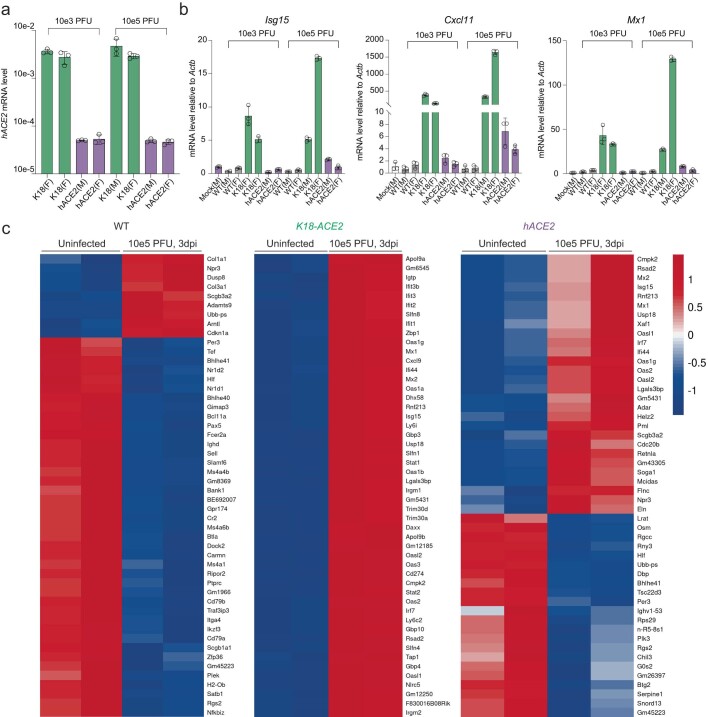
Interferon responses in SARS-CoV-2 infected lungs. (**a**) Human *ACE2* expression level analysis by RT-qPCR, human *ACE2* expression levels were normalized *Actb*. Bars represent mean ± SD of three technical replicates. F, female, M, male. (**b**) RT-qPCR analysis of three interferon-stimulated genes, *Isg15*, *Cxcl11*, *Mx1* in SARS-CoV-2 infected lungs at 3 dpi. Expression was normalized to *Actb* and to an uninfected control. Bars represent mean ± SD of three technical replicates. F, female, M, male. (**c**) Heatmaps of top 50 differentially expressed genes of wild-type, *K18-hACE2* and *hACE2* infected lungs comparing uninfected lungs. Color scale, z-score.

**Extended Data Fig. 8:**
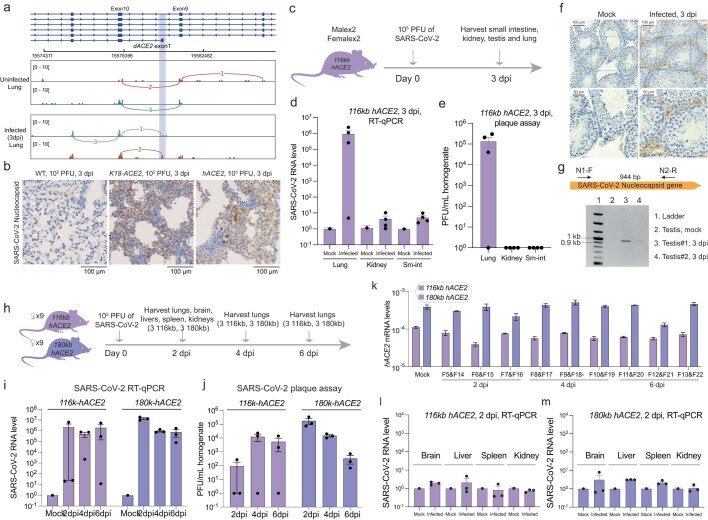
SARS-CoV-2 infection characterization. (**a**) RNA-seq Sashimi plots of SARS-CoV-2 infected (3 dpi) or uninfected lungs. Numbers are read counts spanning exon-exon junction. (**b**) IHC staining of wild-type, *K18-hACE2* and *hACE2* lungs with SARS-CoV-2 nucleocapsid protein antibody (Thermo Fisher Scientific, MA1-7404). Lung sections that are adjacent to H&E staining section (Fig. 5f) were used. (**c**) Two male and two female *116kb-hACE2* mice were intranasally infected with 10^5^ PFU SARS-CoV-2. Lung, kidney, small intestine and testis were harvested at 3 day-post-infection. (**d**) SARS-CoV-2 nucleocapsid gene was detected by RT-qPCR, and normalized to *Actb*, and then normalized to uninfected control. Bars represent mean ± SEM of biological replicates, n = 4 independent mice. (**e**) Infectious viral from lung, kidney and small intestine were quantified by plaque assay. Bars represent mean ± SEM of biological replicates, n = 4 independent mice. (**f**) IHC staining of mock or infected (3 dpi) testes with SARS-CoV-2 nucleocapsid protein antibody (Thermo Fisher Scientific, MA1-7404). (**g**) RT-PCR SARS-CoV-2 nucleocapsid gene fragment from mock or infected (3 dpi) *116kb-hACE2* testes. A 944 bp DNA fragment was amplified from 3 dpi testes and the DNA fragments were loaded in a 1% agarose. (**h**) Nine *116kb-hACE2* and nine *180kb-hACE2* mice were intranasally infected with 10^5^ PFU SARS-CoV-2. Lungs were harvested at 2 dpi, 4 dpi and 6 dpi. Brains, livers, spleens and kidneys were harvested at 2 dpi. (**i**-**j**) Lungs harvested from *116kb-hACE2* and *180kb-hACE2* mice infected with SARS-CoV-2 were analyzed for SARS-CoV-2 nucleocapsid gene expression by RT-qPCR (i), and infectious viral levels by plaque assay (j). SARS-CoV-2 levels were normalized *Actb* and then to an uninfected control. Bars represent mean ± SEM of biological replicates, n = 2 independent mice at each time point. (**k**) Human *ACE2* expression levels in the infected lungs, *hACE2* levels were normalized to *Actb*. Bars represent mean ± SD of three technical replicates. (**l**-**m**) RT-qPCR detection of SARS-CoV-2 nucleocapsid gene in indicated tissues from *116kb-hACE2* (l) and *180kb-hACE2* (m) mice. SARS-CoV-2 levels were normalized *Actb* and then to an uninfected control. Bars represent mean ± SEM of biological replicates, n = 2 independent mice at each time point.

To characterize the susceptibility of *ACE2* mice to SARS-CoV-2, we challenged the *ACE2, K18-hACE2* and wild-type mice intranasally with 10^3^ or 10^5^ plaque-forming units (PFU) of SARS-CoV-2. All mice were euthanized 3 days post-infection (dpi) and viral RNA level in dissected lungs was evaluated by qPCR with reverse transcription (RT–qPCR). As expected, SARS-CoV-2 RNA was undetectable in wild-type lungs; although high levels of SARS-CoV-2 RNA positively correlating with the inoculum dose were detected in *K18*-*hACE2* lungs (Fig. 5a). In the *ACE2* mice, we detected moderate levels of viral RNA in the 10^5^ PFU infection group, and very low amounts in the male *ACE2* mouse of the 10^3^ PFU infection group. We quantified infectious viruses from lung homogenates using a plaque assay (Fig. 5b), and found the levels to be consistent with the result from RT–qPCR. We noted that higher viral RNA levels were detected in lungs from male *K18-hACE2* and *ACE2* mice compared with the female mice, despite identical inoculum dosage. We found no significant difference in *ACE2* expression between males and females (Extended Data Fig. 7a). Notably, *ACE2* mice displayed around 70-fold lower *ACE2* expression in lungs compared with transgenic *K18-hACE2* mice (Extended Data Fig. 7a). The host interferon-stimulated genes *Isg15*, *Cxcl11* and *Mx1* were significantly induced in the *K18-hACE2* mice, and these were also induced in the *ACE2* mice—albeit to a lower degree—mirroring viral levels (Extended Data Fig. 7b). Transcriptional evaluation of SARS-CoV-2-infected lungs revealed a moderate type I/III interferon response in the *ACE2* mice (Fig. 5c), in which the induced genes largely overlap with those induced in *K18-hACE2* mice, but not with those induced in wild-type mice (Fig. 5d, Extended Data Fig. 7c and Supplementary File 2). Notably, RNA-sequencing analysis showed an increase in the amount of *dACE2* transcript in SARS-CoV-2-infected *ACE2* mice (Extended Data Fig. 8a), consistent with data from humans^26^. We confirmed the upregulation of the *dACE2* isoform in other infected *ACE2* mice using RT–qPCR (Fig. 5e). Histopathological examination of infected lung sections revealed that both *K18-hACE2* and *hACE2* mice developed pneumonia, evidenced by monocyte infiltration, but *hACE2* mice displayed substantially milder lesions of alveolar epithelial cells (Fig. 5f and Supplementary Fig. 3). Corresponding immunohistochemistry showed strong SARS-CoV-2 nucleocapsid protein staining surrounding alveolar cells in both models (Extended Data Fig. 8b).

To determine whether SARS-CoV-2 infects other mouse organs, we collected small intestine, kidney and testes at 3 dpi after 10^5^ PFU intranasal infections. We did not detect viral RNA or infectious virus in small intestine or kidney (Extended Data Fig. 8c–e). Immunohistochemistry of infected testes showed the presence of SARS-CoV-2 nucleocapsid protein primarily on the membrane of Leydig cells, which produce testosterone in male mice (Extended Data Fig. 8f). RT–PCR confirmed the presence of SARS-CoV-2 nucleocapsid mRNA in the infected testes (Extended Data Fig. 8g). Unlike in patients with severe COVID-19, where SARS-CoV-2 was mostly detected in germ cells in seminiferous tubules^40^, the virus did not enter the seminiferous tubules in the *ACE2* mice, possibly owing to virus clearance by immune surveillance in these immunocompetent mice.

Given that the 180 kb-*ACE2* model expresses 3- to 5-fold more *ACE2* mRNA in lung compared with the 116 kb-*ACE2* model (Extended Data Fig. 6c), we tested whether this difference contributed to the outcome of SARS-CoV-2 infection. We infected 116 kb-*ACE2* and 180 kb-*ACE2* models with SARS-CoV-2 and collected total RNA and homogenate from lungs at 2, 4 and 6 dpi (Extended Data Fig. 8h). All 180 kb-*ACE2* mice were readily infected starting at 2 dpi, and viral levels decreased over time. By contrast, despite identical infection conditions, virus was not detectable in some of the 116 kb-*ACE2* lungs (Extended Data Fig. 8i,j). Consistent with our previous results in uninfected mice (Extended Data Fig. 6c), we detected higher *ACE2* mRNA levels in the infected 180 kb-*ACE2* lungs (Extended Data Fig. 8k). We speculate that differences in infection kinetics result from the higher *ACE2* expression levels in 180 kb-*ACE2* mice. Additionally, viral RNA was undetectable in brain, liver, spleen and kidney of 180 kb-*ACE2* mice (Extended Data Fig. 8l,m).

Human COVID-19 is a complex disease with diverse manifestations and outcomes reflecting the age, health status, immune status and genetic makeup of infected individuals. We thus tested whether *ACE2* mice could be used to better model human SARS-CoV-2 infection compared with *K18-hACE2* mice, which succumb to SARS-CoV-2 within 10 days^19^ and are thus unable to recapitulate medium-term and long-term effects of viral infection. We infected *ACE2* and *K18-hACE2* mice with 10^5^ PFU of SARS-CoV-2, and monitored their weight and survival over the course of 14 days. All *ACE2* mice survived to the end without obvious sickness. By contrast, *K18-hACE2* mice had significantly reduced mobility at 5 dpi; 4 out of the 5 mice died at 6 dpi; and the remaining mouse died at 8 dpi (Fig. 5g). Body weight measurements showed that *K18-hACE2* mice lost a significant amount of body weight before they died, whereas the *ACE2* mice did not lose any weight over the course of the experiment (Fig. 5h). Measurements of antiviral humoral immune response using enzyme-linked immunosorbent assay (ELISA) showed evidence of antibodies that recognized the spike trimer in sera from *ACE2* mice at 14 dpi (Fig. 5i). Collectively, these data suggest that *ACE2* mice can recover from SARS-CoV-2 infection, and are thus useful for modelling aspects of human COVID-19 pathophysiology.

**Extended Data Fig. 9:**
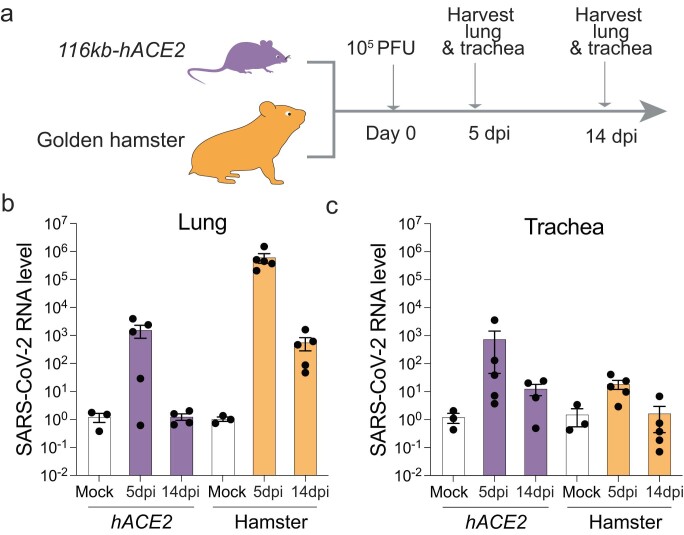
Comparing SARS-CoV-2 infection between hACE2 mice and golden hamster. (**a**) Schematic of the longitudinal SARS-CoV-2 infection experiment for *116* *kb* mice and wild-type golden hamsters. (**b-c**) RT-qPCR analysis of SARS-CoV-2 nucleocapsid gene in lung (b) and trachea (c) of *116* *kb* mice and hamsters. *hACE2* mice, n = 5 (5 dpi), 4 (14 dpi). Hamsters, n = 5 (5 dpi), 5 (14 dpi). SARS-CoV-2 RNA level was normalized *Actb* and to uninfected control. Bars represent mean ± SEM of biological replicates.

The golden hamster (*Mesocricetus auratus*) is a commonly used rodent model for studying respiratory virus infections^41^. However, such studies are limited by a lack of genetic tools, and a limited repertoire of hamster mutants used to model comorbidities. We tested whether *ACE2* mice were similar to hamsters in terms of SARS-CoV-2 susceptibility. We set up a longitudinal infection, including collection of lungs and tracheas at 5 and 14 dpi (Extended Data Fig. 9a). SARS-CoV-2 viral RNA was detected at 5 dpi in lung of *ACE2* mice and hamsters; the level of viral RNA was lower in *ACE2* mice, and was diminished significantly by 14 dpi (Extended Data Fig. 9b). In hamster trachea, viral RNA levels increased slightly at 5 dpi (Extended Data Fig. 9c), probably owing to a lack of *Ace2* expression in hamster tracheal epithelial cells^42^. By contrast, higher levels of viral RNA were detected in the trachea of a subset of *ACE2* mice, consistent with previous results in human patients^43^. Thus, the *ACE2* GEMM has a milder but comparable disease course to golden hamster in lungs, and perhaps more human-like susceptibility to tracheal infection.

## Biallelic *TMPRSS2* humanization in *ACE2* mouse ES cells

Following attachment of SARS-CoV-2 to ACE2, cleavage of the spike S2 subunit by transmembrane protease serine 2 (TMPRSS2) on the host cell membrane^44^ is crucial for enabling virus–cell membrane fusion. Co-expression of *ACE2* and *TMPRSS2* in lung epithelial cells is required for effective infection^45^. We therefore hypothesized that genomically humanizing *TMPRSS2* in *ACE2* mice would better recapitulate human-specific physiological expression patterns in mice, improving the accuracy of COVID-19 modelling in mice. In addition, humanizing *TMPRSS2* may facilitate the development of therapies targeting TMPRSS2 activity, since its physiological role is not clearly defined, and *Tmprss2*-knockout mice exhibit no phenotypic abnormalities^46^.

**Fig. 6:**
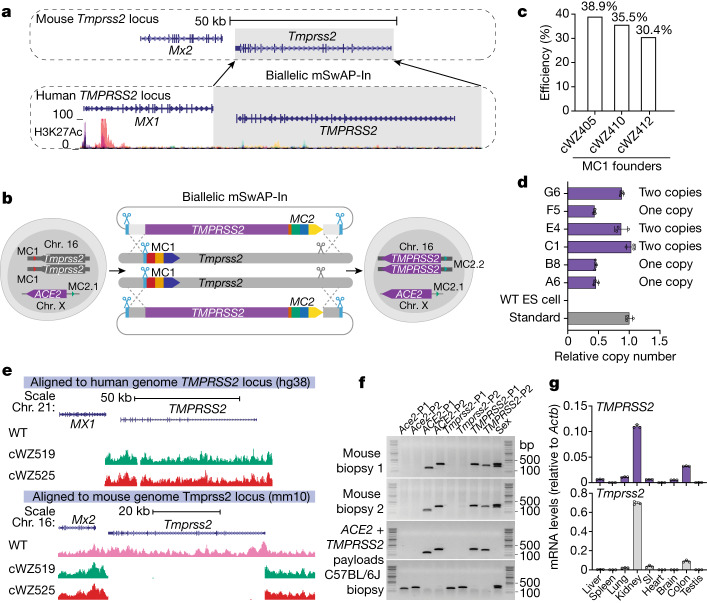
Serial, biallelic humanization of *TMPRSS2* in *ACE2* mouse ES cells. **a**, Schematic of *TMPRSS2* humanization design. Top, *Tmprss2* gene locus; grey box highlights the region replaced by human *TMPRSS2*. Bottom, human *TMPRSS2* locus; shading highlights the humanization region. The 3′ end of *MX1* was defined as the left boundary; for the right boundary, sufficient *TMPRSS2* upstream genomic sequence was used to include a putative enhancer. **b**, Schematic workflow for *TMPRSS2* biallelic humanization in *ACE2* mouse ES cells. **c**, Success rate of biallelic humanization in three MC1 mouse ES cell founder lines determined by genotyping PCR: cWZ405 (*n* = 76), cWZ410 (*n* = 81) and cWZ412 (*n* = 13). **d**, *TMPRSS2* copy number determination by qPCR. Copy number determined as in Fig. 3e. WT, wild type. **e**, Sequencing coverage of *TMPRSS2* mouse ES cell clones. Reads mapped to hg38 (top) and mm10 (bottom). **f**, A double-humanized GREAT-GEMM with both *ACE2* and *TMPRSS2* was established via tetraploid complementation. Sex: male, two bands (X and Y); female, one band (X only). **g**, Top, *TMPRSS2* expression pattern in double-humanized *ACE2* and *TMPRSS2* mouse. Bottom, mouse *Tmprss2* expression pattern in *ACE2-*only humanized mouse. Data are mean ± s.d. of three technical replicates.

**Extended Data Fig. 10:**
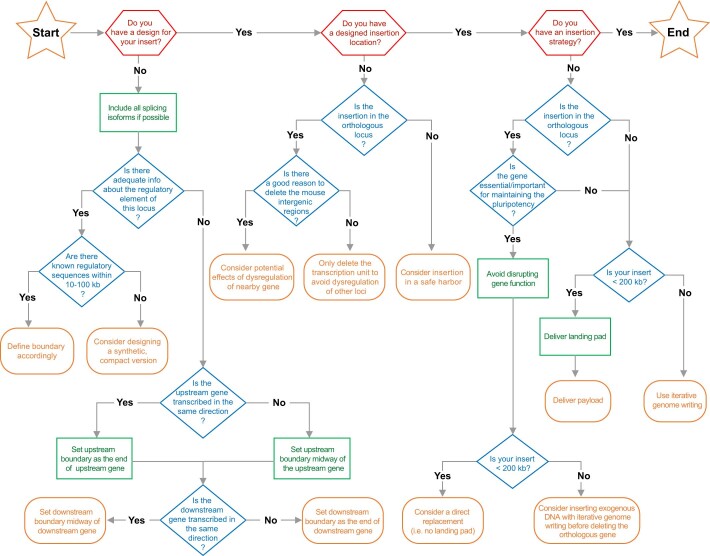
Generalized design scheme for mouse gene humanization using mSwAP-In. A logic flowchart guides mSwAP-In users to design mouse gene humanization.

**Extended Data Fig. 11:**
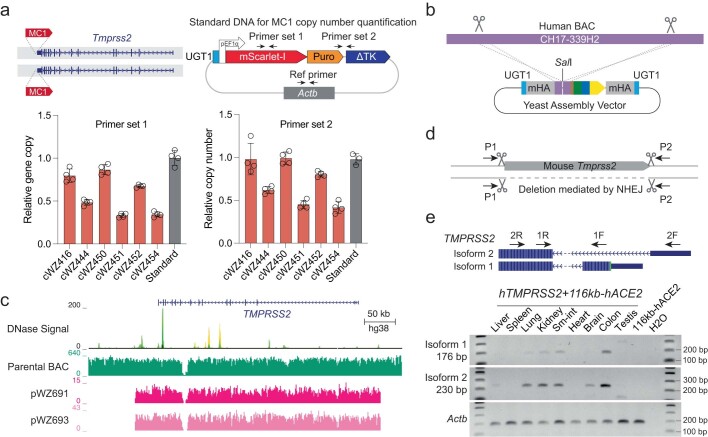
*TMPRSS2* humanization and characterization. (**a**) Biallelic marker cassette 1 insertion and copy number quantification. A reference plasmid with one copy of marker cassette 1 and one copy of mouse *Actb* gene serves as the standard. Two pairs of primers targeting different regions of MC1, one pair of primer targeting *Actb* were used for qPCR. MC1 copy number was normalized to *Actb*, and then normalized to the standard DNA. Bars represent mean ± SD of three technical replicates. (**b**) Human *TMPRSS2* mSwAP-In payload assembly strategy, scissors indicate Cas9-gRNA cutting sites in vitro. (**c**) UCSC browser shows sequencing verification of the *hTMPRSS2* payload, reads were mapped to the hg38 reference. (**d**) Strategy for distinguishing truly biallelic *TMPRSS2* integration from hemizygous integration. P1 and P2 are two primers that bind outside of the CRISPR-Cas9 cutting sites (500 bp to 1 kb away). (**e**) Two human *TMPRSS2* splice isoforms were detected by RT-qPCR in various mouse tissues.

To test the feasibility of serially editing mouse ES cells using mSwAP-In, we explored the possibility of overwriting both *Tmprss2* alleles simultaneously using mSwAP-In, exploiting a first-generation generic design scheme (Extended Data Fig. 10). We designed and built reagents to insert human *TMPRSS2*, replacing *mTmprss2* (Fig. 6a). To do so, MC1 was biallelically inserted downstream of *Tmprss2* in the 116 kb-*ACE2* mouse ES cells (Extended Data Fig. 11a). An 80-kb human *TMPRSS2* mSwAP-In payload was assembled (Extended Data Fig. 11b,c), and delivered into biallelic MC1 insertion founder lines. Both *Tmprss2* alleles were replaced by the human payload, resulting in biallelic *TMPRSS2* humanization (Fig. 6b) at 30%–40% efficiency (Fig. 6c). Copy number analysis confirmed that around 50% of those clones had two copies of *TMPRSS2* (Fig. 6d). Clones with one copy of *TMPRSS2* exhibited deletion of one *Tmprss2* allele (Extended Data Fig. 11d). Capture sequencing and subsequent genotyping of mouse biopsies confirmed the accuracy of *TMPRSS2* humanization (Fig. 6e,f). *ACE2* and *TMPRSS2* double-humanized mice were obtained via tetraploid complementation, demonstrating that an additional round of biallelic mSwAP-In engineering did not impair mouse development from mouse ES cells. Backcrossing *ACE2* and *TMPRSS2* double-humanized males resulted in 100% heterozygous *TMPRSS2* progeny (data not shown), confirming that *TMPRSS2* humanization was biallelic. We detected *TMPRSS2* expression in liver, lung, kidney, small intestine, brain and colon, similar to the pattern of *Tmprss2* expression in *ACE2-*only humanized mice (Fig. 6g). Two *TMPRSS2* splice isoforms^47^ (transcript 1 and 2) were detected in various tissues (Extended Data Fig. 11e), indicating that *TMPRSS2* is properly transcribed and spliced in mouse, similar to *ACE2* (Fig. 4d,e).

## Discussion

Understanding mammalian genomes requires exploration from distinct perspectives. Advanced sequencing technologies have revealed the complex blueprints of many vertebrates. Here, to directly probe the roles of regulatory components and genome polymorphisms, we provide a strategy to reliably overwrite large stretches of native mammalian genomic segments with carefully designed synthetic DNAs or cross-species gene counterparts. Mammalian genome writing is ideal for introducing tens to hundreds of edits through de novo synthesis—which would otherwise be extremely difficult, if not impossible, to engineer with traditional genome-editing approaches such as CRISPR—while maintaining cells’ developmental potential throughout multiple editing rounds. The iterative nature of mSwAP-In genome writing overcomes size limitations of DNA delivery, paving the way for eventual writing of megabase-sized synthetic DNAs. The combination of positive and negative selection ensures on-target integration of payloads. In conjunction with targeted capture sequencing, clones with undesirable genomic outcomes (for example, integration of plasmid backbones, co-transfected plasmids or payload structural variants) can be identified and eliminated, reducing experimental bias.

Although we have demonstrated that mSwAP-In can be used to deliver diverse large DNAs to mouse ES cells, we believe that mSwAP-In can be generalized to other mammalian species, provided that HR in the species has similar efficiency to that in mouse ES cells.

Mice are commonly used as pre-clinical models, but human diseases are often not fully recapitulated owing to evolutionary differences. Genetically humanizing complete mouse loci by in situ replacement provides a means to more accurately recapitulate disease as human-specific spatiotemporal regulation and splicing are often preserved. The high efficiency of mSwAP-In combined with the speed with which transgenic animals can be produced using tetraploid complementation enables fast production of informative GREAT-GEMMs.

We generated a genomically humanized *ACE2* mouse model with mSwAP-In. In contrast to existing humanized *ACE2* models, the *ACE2* expression level and distribution in these mice more closely resembled those seen in humans (Fig. 4). These mice are readily infected with SARS-CoV-2, display relatively mild disease symptoms without mortality, and produce a humoral antiviral response, resembling outcomes observed in healthy young humans. Our humanized *ACE2* mouse model is likely to be a valuable platform for studying long-term effects of COVID-19 in vivo. Mortality and more severe symptoms are common in elderly and comorbid individuals. The *ACE2* mice used here were relatively young (10–15 weeks old) and healthy, corresponding to young people with mild or minimal COVID-19 symptoms. Infection experiments using older *ACE2* mice or combining *ACE2* with existing mouse models of conditions such as diabetes or obesity, may inform the understanding of severe COVID-19. Finally, we leveraged the biallelic genome writing power of mSwAP-In to create homozygous *TMPRSS2* and *ACE2* double-humanized mice, demonstrating the usefulness and speed of mSwAP-In for producing double-humanized GREAT-GEMMs.

## Methods

### BAC plasmids

Human (CH17-203N23, CH17-449P15 and CH17-339H2) and mouse (RP23-51O13, RP23-75P20 and RP23-204E8) BACs were purchased from BACPAC Resources Center. Yeast–bacterium shuttle vector pLM1050 was modified by L. Mitchell based on a previous study^28^. pWZ699 was constructed by inserting a cassette containing pPGK-ΔTK-SV40pA transcription unit and the *Actb* gene into the NotI site of pLM1050. Marker cassette 1 donor plasmids for *synTrp53* and *ACE2* loci were constructed using Gibson assembly of MC1 and two homology arms into pUC19 vector. Left and right homology arms of ~750 bp were amplified from the corresponding BACs. When using microhomology-mediated end joining for MC1 insertion, 20-bp microhomology arms were carried on primers. pX330 plasmid was purchased from Addgene (42230).

### Mammalian cell lines and yeast strain

The C57BL/6 J mouse ES cell line (MK6) was obtained from NYU Langone Health Rodent Genetic Engineering Core.  MK6 and its derivatives described here were used extensively. Many of its loci were sequenced in our laboratory. It was tested for mycoplasma contamination and was found to be negative. Both feeder-dependent and feeder-independent culture conditions were used for different purposes in this study. The mouse ES cell medium for feeder-dependent condition consists of 85% (v/v) KnockOut DMEM (Fisher Scientific, 10829018), 15% (v/v) Fetal Bovine Serum (Hyclone, SH30070.03), 0.5 mg ml^−1^ Penicillin-Streptomycin-Glutamine (Gibco, 10378016), 7 μl 2-mercaptoethanol (Sigma-Aldrich, M6250), 0.1 mM MEM Non-Essential Amino Acids (Gibco, 11140050) and 1,000 U ml^−1^ LIF (EMD Millipore, ESG1107). Tissue culture treated plates were first coated with 0.1% gelatin solution (EMD Millipore, ES-006-B), followed by seeding 7.5 × 10^4^ cm^−2^ of mouse embryonic fibroblast (MEF) cells (CellBiolabs, CBA-310) in MEF medium (DMEM (Gibco, 11965118), 10% Fetal Bovine Serum (GeminiBio, 100–500), 0.1 mM MEM Non-Essential Amino Acids, 2 mM l-glutamine, 1% penicillin-streptomycin). Mouse ES cells were plated on the MEF monolayer. Feeder-independent medium consisted of 80% of 2i basal medium supplement with 3 µM CHIR99021 and 1 µM PD0325901, 20% of feeder-dependent mouse ES cell medium (mentioned above). Tissue culture treated plates were coated with 0.1% gelatin solution before use. All cells were grown in a humidified tissue culture incubator at 37 °C supplied with 5% CO_2_. VeroE6 cells (kidney epithelial cells from female African green monkey, ATCC, CRL-1586) were cultured in 12-well plates with DMEM supplemented with 4% FBS, 1% penicillin-streptomycin-neomycin and 0.2% agarose (Lonza, 50100). BY4741 yeast strain was used for all the payload assemblies.

### Virus

SARS-CoV-2 strain USA-WA1/2020 (NR-52281) was obtained from BEI Resources, NIAID, NIH. SARS-CoV-2 viruses were expanded in VeroE6 cells^41^. Collected viruses were purified with an Amicon Ultra-15 Centrifugal filter unit (Millipore Sigma). The SARS-CoV-2 virus stock titre was determined by performing a plaque assay in VeroE6 cells.

### Animals

Engineered mouse ES cells were either injected into C57BL/6J-albino (Charles River Laboratories, strain no. 493) blastocysts, or B6D2F1/J (Jackson laboratories, strain no. 100006) tetraploid blastocysts for mice production. Mice were housed in NYU Langone Health BSL1 barrier facility. Wild-type C57BL/6 J (strain no. 000664) and *K18*-*hACE2* (strain no. 034860) mice were obtained from The Jackson laboratory. Golden hamsters were obtained from Charles River Laboratories (strain no. 049). Ten- to-fifteen-week-old mice and ten- to twelve-week-old hamsters were transferred to the NYU Langone Health BSL3 facility for SARS-CoV-2 infection. All mice were settled for at least two days prior to infection. Similar aged mice or hamsters were randomly grouped into different cages. Animal sample sizes were chosen to enable significant statistical power while minimizing unnecessary wastage. Animals were housed in 12 h light:12 h dark cycle, ambient temperature and humidity condition. All experimental procedures were approved by the Institutional Animal Care and Use Committee (IACUC) at NYU Langone Health.

### Payload DNA assembly and preparation

Two approaches were used for payload DNA assembly in this study. For synthetic *synTrp53* and its subsequent 40 kb, 75 kb and 115 kb payloads, DNA fragments ranging from 3 kb to 5 kb with 40–100 bp terminal homologies were amplified from mouse BAC RP23-51O13 using Q5 polymerase (NEB, M0491L). Approximately equal amount (100 ng) of each PCR fragment, mixed with 50 ng of each linker fragment for bridging vector and insert and 20 ng linearized pLM1050 vector were co-transformed into yeast for assembly. For *ACE2* and *TMPRSS2* payloads, CH17-203N23, CH17-449P15 and CH17-339H2 BACs were extracted by using a NucleoBond Xtra BAC kit (Takara, 740436.25). Approximately 1 μg of BAC DNA was digested with 30 nM of sgRNAs (IDT), and 30 nM recombinant Cas9 nuclease (NEB, M0386S) at 37 °C for 2 h. 1 μl of 20 mg ml^−1^ proteinase K was added to the digestion reaction for 10 min at room temperature. Digested BAC and SalI-linearized acceptor vector (Fig. 3b and Extended Data Fig. 11b) were co-transformed into yeast for assembly. Yeast cells were cultured on SC–Leu plates at 30 °C for 3 days. Yeast colony containing correct payload was identified by screening all novel junctions between each two fragments. To assemble the 180 kb-*hACE2* payload, an *URA3* gene was inserted in front of the MC2 of the 116 kb-*ACE2* payload. The 64-kb *ACE2* region of interest was released from CH17-449P15 BAC by in vitro Cas9–gRNA digestion. A plasmid expresses Cas9 and gRNA targeting *URA3* in yeast was co-transformed with the 64 kb *ACE2* fragment into BY4741 strain containing 116 kb-*ACE2* payload. Yeast cells were selected with 5-fluoroorotic acid for successful insertion of the 64 kb *ACE2* fragment. Payload DNAs were isolated from yeast by using a yeast plasmid miniprep kit (Zymo Research, D2001), eluted in 30 μl of TE. Two microlitres of yeast miniprep DNA was used for electroporation into EPI300 *E. coli* strain (Lucigen, EC300150). *E. coli* colonies containing payload DNAs were grown in 5 ml LB medium supplemented with 50 μg ml^−1^ kanamycin overnight, and diluted at 1:100 ratio into 250 ml LB supplemented with kanamycin (50 μg ml^−1^) and 1× copy number induction solution (Lucigen, CCIS125). Payload DNA was isolated from *E. coli* by using a NucleoBond Xtra BAC kit (Takara, 740436.25) for delivery into mouse ES cells. Primers used for payload assembles are listed in Supplementary File 3.

### BAC and payload DNA sequencing library construction

Concentration for BACs and assembled payload DNAs was quantified by using a Qubit dsDNA HS kit (Thermo Fisher, Q32854), Approximately 100 ng DNA was used for the library construction using the NEBNext Ultra II FS DNA library prep kit (E7805). AMPure XP beads (Beckman Coulter, A63881) were used for DNA purification on a magnetic stand. DNA libraries were loaded on a ZAG DNA analyser (Agilent) for quality control. DNA libraries were sequenced on an Illumina NextSeq 500.

### Sequencing data processing

Sequencing reads were demultiplexed using bcl2fastq v2.20, and subsequently trimmed using Trimmomatic v0.39. Trimmed reads were aligned to references using BWA v0.7.17. Duplicates were marked using samblaster v0.1.24. Coverage depth tracks and quantification was generated using BEDOPS v2.4.35. Sequencing data were visualized using UCSC genome browser. The sequencing processing pipeline is available at https://github.com/mauranolab/mapping.

### Pulse-field gel electrophoresis

Payload DNAs were linearized using a single-cut restriction enzyme, followed by heat inactivation as recommended by the manufacturer. Two-hundred nanograms of digestion product was loaded into a 1% low-melting point agarose gel. Lambda-PFG ladder (NEB, N0341S) or lambda DNA-Mono cut mix (NEB, N3019S) were used as ladders. CHEF Mapper XA System (Bio-Rad), auto-algorithm was used for electrophoresis. Agarose gel was first stained with 0.5 μg ml^−1^ ethidium bromide in deionized water for 30 min, and then de-stained with deionized water for 30 min before imaging on a ChemiDoc MP imaging system (Bio-Rad).

### Crystal violet staining

Mouse ES cell clones grown on gelatin-coated plates were washed with PBS once, then fixed in 4% (w/v) formaldehyde for 15 min at room temperature followed by 2 rounds of washing with PBS. 0.1% (diluted with 10% ethanol) crystal violet (Sigma-Aldrich, V5265) dye was used to stain the mouse ES cell colonies for 20 min at room temperature followed by 3 rounds of washing with water. Plates were air-dried at room temperature before counting the colony number.

### Flow cytometry

*synTrp53* and wild-type *Trp53* mouse ES cells were cultured under feeder-independent condition. Cells were grown in medium containing 250 nM doxorubicin (Tocris, 2252) for desired period. After the doxorubicin treatment, mouse ES cells were trypsinized and stained with Hoechst33342 (Invitrogen, H3570) for 30 min at room temperature for DNA content-based cell cycle analysis, or stained with annexin V conjugated with 680 fluorophores (Invitrogen, A35109) for 15 min at room temperature for apoptosis analysis. Stained cells were analysed using a SONY SH800s instrument. Data were analysed using SONY SA3800, SH800s and FlowJo software.

### Nucleofection

Depending on the culture conditions, 10-cm tissue culture dishes were pre-coated with either 0.1% gelatin (EMD Millipore, ES-006-B) or mitomycin-treated MEF feeder cells. Mouse ES cells were trypsinized with 0.25% Trypsin-EDTA (Gibco, 25200056) at 37 °C for 6 min. Cell number was determined by hemocytometer. Approximately 3 million of mouse ES cells were washed with DPBS (Gibco, 14190144) and pelleted by centrifugation at 300*g* for 5 min at room temperature. A total of 10 μg DNA mixture containing payload DNA and Cas9–gRNA plasmid(s) (Supplementary Table 3) was used for the nucleofection. Nucleofection solutions and cuvette were from Mouse ES Cell Nucleofector kit (Lonza, VPH-1001). Nucleofector (Lonza 2b) A-023 program was used to deliver the DNA mixture into mouse ES cells. Nucleofected mouse ES cells were plated onto pre-coated 10-cm dishes, and cultured in 37 °C, 5% CO_2_ humidified incubator.

### Mouse ES cell colony picking and PCR screening

Mitotically inactivated MEFs were pre-seeded in a 96-well tissue culture plate (Corning, 3595) in MEF medium 1 day before colony picking. The next day, MEF medium was swapped to 100 μl per well of ES medium at least 2 h before use. The 10-cm plates containing mouse ES cell colonies were washed with DPBS once, and refilled with 10 ml DPBS. Mouse ES cell colonies were aspirated with 10 μl of DPBS using a P20 pipette, and transferred to an empty round bottom low-retention 96-well plate. Thirty-five microlitres per well of accutase (Gibco, A1110501) was added to the mouse ES cell colonies for dissociation at 37 °C for 9 min. One-hundred microlitres per well of ES medium was used to neutralize the trypsinization reaction. Mouse ES cells were singularized by at least 20 times of gentle pipetting. One-hundred microlitres of the cell suspension was transferred to a gelatin-coated 96-well plate prefilled with 100 μl of ES medium. The rest of cell suspension (~40 μl) was transferred to the 96-well MEF plate prefilled with 100 μl of ES medium. ES cell medium was refreshed daily until the feeder-independent plate becomes >50% confluent. Mouse ES cells from feeder-independent plate were trypsinized and 10% cells were passaged to a new gelatin-coated plate for proliferation, 90% of cells were transferred to a PCR plate. Mouse ES cells in the PCR plate were spun down at 300*g* for 5 min, and supernatant was discarded. Cell pellets were resuspended with 30 μl of lysis buffer (0.3 mg ml^−1^ proteinase K in TE). Mouse ES cells were lysed on a thermal cycler using 37 °C 1 h, 98 °C 10 min, 16 °C keep program. One microlitre of mouse ES cell lysate was used as template in a 10-μl PCR reaction.

### Digital PCR for copy number determination of human *ACE2*

Genomic DNA of mouse ES cells was extracted by using a QIAamp DNA mini kit (QIAGEN, 51306). Approximately 500 ng of mouse ES cell gDNA and payload DNA containing the *Actb* gene on the backbone were digested with *Eco*RI (NEB, R3101S) at 37 °C for 2 h. Fifty nanograms digested mouse ES cell gDNA and 1 pg digested payload DNA were used for qPCR analysis. For *synTrp53* mouse ES cells, a wild-type mouse ES cell gDNA sample was used as normalization control. SYBR Green Master Mix (Roche, 04887352001) was used for the qPCR reaction on a LightCycler 480 instrument. Copy number was normalized to *Actb* containing payload (for *ACE2* and *TMPRSS2* clones) or wild-type mouse ES cells (for *synTrp53* clones).

### Mouse ES cells capture sequencing library construction

A total of 1–3 million feeder-independent mouse ES cells were collected for genomic DNA extraction using a QIAamp DNA Mini Kit (QIAGEN, 51306). Genomic DNA concentration was determined by using a Nanodrop spectrophotometer. Approximately 1 μg genomic DNA was used for DNA library construction with a large fragment size protocol (NEBNext Ultra II FS). Final DNA library concentration was measured by using a Qubit dsDNA HS assay kit (Invitrogen, Q32851). For the *synTrp53* mouse ES cells, capture bait comprises RP23-51O13, MC1, MC2 and pX330 DNAs. For *ACE2* humanized mouse ES cells, capture bait comprises CH17-203N23, CH17-449P15, RP23-75P20, MC1, MC2 and pX330 DNAs. Bait DNA mixture was labelled with Biotin-16-dUTP (Roche, 11431692103) using a nick translation kit (Sigma-Aldrich, 10976776001). The capture was performed as previously described^15^. In brief, biotinylated bait DNA mixture was prehybridized, and mixed with DNA library samples at 65 °C for 16 to 22 h. Captured DNA was purified using Streptavidin C1 beads (Invitrogen, 65002) and amplified using KAPA Hi-Fi HotStart PCR kit (Roche, KK2602). After a final step of DNA cleanup, captured libraries were sequenced on an Illumina NextSeq 500 using a 75 cycles kit.

### *Trp53* amplicon-seq

PCR was used to amplify the six *Trp53* recoded codon regions and simultaneously tag each template molecule with terminal UMIs. The total targeted region was divided into three amplicons with lengths of 108 bp, 76 bp and 132 bp to ensure accurate sequencing (Supplementary Table 4). The first section of both tailed primers targets the priming site, followed by the UMI on the reverse primer, consisting of a total of 10 randomized nucleotides which results in a total of more than 10^6^ unique UMI tags. The primer termini consist the Illumina sequencing adapter sequences. One cycle of PCR reaction was performed to introduce the UMI to each copy of the 500 ng original genomic DNA molecule. The extension was carried by KAPA-HiFi HotStart polymerase and 200 nM reverse primer. Thermal cycling parameters were as follows: 5 min for pre-incubation at 95 °C, followed by 60 °C annealing for 1 min and 72 °C elongation for 10 min. Two additional rounds of PCR were performed to sequentially amplify the region of interest and add sequencing indexes and Illumina sequencing adapters. For the amplicon PCR, all the UMI-tagged template molecules were added to 50-μl reaction containing KAPA-HiFi HotStart and 200 nM of each primer. Thermal cycling parameters were as follows: 5 min for pre-incubation at 95 °C, followed by followed by 23–26 amplification cycles (cycle number corresponds to half of maximum fluorescent intensity) of 15 s at 95 °C, 15 s at 65 °C and 30 s at 72 °C. The PCR product was purified using a SPRI beads (0.8×) cleanup. For the barcoding PCR, 1:20 of the amplicon PCR sample was added to the reaction containing KAPA-HiFi HotStart and 200 nM of each primer. Thermal cycling parameters were as follows: 5 min for pre-incubation at 95 °C, followed by followed by 8–12 amplification cycles (cycle number corresponds to half of maximum fluorescent intensity) of 15 s at 95 °C, 15 s at 71 °C and 30 s at 72 °C. The PCR product was purified using a SPRI beads (0.8×) cleanup and quantified using Qubit HS DNA kit. Amplicon libraries were sequenced using paired ends 150 bp method on a NovaSeq instrument. Amplicon reads pairs with more than 75% of G bases were removed, and poor-quality reads were filtered out using fastp^48^ with options “-A -G -q 30 -u 15”. UMI sequences were extracted using UMI-tools v1.0.1 (ref. ^49^) including the option “--quality-filter-threshold=30” from reads with no mismatch against the primer sequence. UMIs were deduplicated using a directed adjacency approach based on UMI-tools and counted the total number of UMIs supporting each base substitution against the template.

### RT–qPCR

Mouse tissues were dissected and homogenized using a pellet pestle (Fisher Scientific, 12141364). mouse ES cells were lysed using QIAshredder (QIAGEN, 79654) Total RNA was extracted using a RNeasy kit following vendor’s instructions (QIAGEN, 74136). Approximately 1 μg of total RNA was used for reverse transcription (Invitrogen, 18091050). One microlitre of 1:10 diluted cDNA was used in a 10-μl SYBR Green (Roche, 04887352001) qPCR reaction on a LightCycler 480 instrument (Roche). Primers used for RT–qPCR are listed in Supplementary Table 5.

### CUT&RUN

Testes were dissected from ~36-week-old male mice. After washing in a 6-cm dish with DPBS, testes were cut into small pieces to expose the seminiferous tubules. Seminiferous tubules were transferred to a 15-ml tube containing 5 ml dissociation buffer (DMEM with 10% FBS, 1% penicillin-streptomycin, 0.25 mg ml^−1^ collagenase–dispase (Roche, 10269638001)) for 30 min incubation at 37 °C. Tubes were inverted every 5 min. Seminiferous tubule fragments were collected and washed with PBS by centrifugation at 300*g* for 5 min at room temperature. Seminiferous tubule fragments were passed through a 70-μm cell strainer, and then washed with DPBS twice. Testicular cell density and viability were evaluated by using an automated Countess cell counter. Five-hundred thousand testicular cells were used for each CUT&RUN reaction by following vendor’s instructions (EpiCypher, 14–1048). In brief, cells were bound to activated ConA beads at room temperature for 10 min. H3K4me3, H3K27ac and negative control (IgG) antibodies were incubated with cells on a nutator at 4 °C overnight. The next day, tubes were placed on a magnet and supernatant was discarded. Cells were permeabilized with buffer containing 0.01% digitonin. Then fusion of proteins A and G to micrococcal nuclease (pAG-MNase) was added to the tubes, and activated by 2 mM CaCl_2_ for digestion 2 h at 4 °C. *E. coli* DNA was spiked in after the pAG-MNase digestion, and DNA was purified using a DNA cleanup column. Sequencing libraries were prepared using the NEBNext Ultra II DNA Library Prep Kit (E7645L). Libraries were sequenced using a 75 cycles kit on Illumina NextSeq 500.

### ATAC–seq

Small intestines were collected from approximately 25-week-old mice. After washing with DPBS, intestines were opened and spread on a bibulous paper. Following 2 washes using DPBS, the intestines were cut into small pieces using a blade, and transferred to 10 ml dissociation buffer (DMEM with 5% FBS, 1% penicillin-streptomycin, 0.25 mg ml^−1^ collagenase–dispase (Roche, 10269638001), 0.25 U ml^−1^ DNaseI (Thermo Scientific, EN0521), 8 mM EDTA, 0.5 mM DTT) for 30 min incubation at 4 °C with gentle shaking. Tissue fragments were collected by removing the supernatant after settling down at room temperature. Ten millilitres wash buffer (DMEM with 5% FBS, 1% penicillin-streptomycin) was added to the tissue fragment pellet, and followed by firmly shaking up and down 8 times. After tissue fragment settled, supernatant containing crypts was transferred to a new 15-ml tube for centrifugation at 300*g* for 5 min at 4 °C. Crypts were resuspended in 1 ml ACK lysis buffer (Gibco, A1049201) for 3 min incubation at room temperature. Four millilitres of wash buffer was added to stop the lysing, crypts were collected by centrifugation at 300*g* for 5 min at 4 °C. Crypts were digested with 1 ml 0.25% trypsin-EDTA (Gibco, 25200056) at 37 °C for 5 min, and the digested was stopped by adding 4 ml wash buffer. Crypts were passed through a 70-μm cell strainer, and intestinal cells were washed in cold PBS twice. Intestinal cell number and viability were evaluated by using an automated cell counter. Approximately 50,000 intestinal cells were collected and washed once with cold PBS at 500*g* for 5 min, 4 °C. Cell pellet was resuspended in 50 μl cold lysis buffer (10 mM Tris-HCl, pH 7.4, 10 mM NaCl, 3 mM MgCl_2_, 0.1% IGEPAL CA-630), and immediately spun down at 500*g* for 10 min, 4 °C. Tn5 transposase was used for the tagmentation reaction (Illumina, 20034197) at 37 °C for 30 min. Fragmented DNAs were purified using a cleanup column (Zymo Research, D4013) and eluted in 10 μl water. All eluted DNA was used as template for a 10 cycles PCR using KAPA-HiFi polymerase in a 50-μl reaction. Library DNA was purified using 1.8× SPRI beads, and sequenced using a 75-cycle kit on the Illumina NextSeq 500.

### In vivo SARS-CoV-2 infection

C57BL/6 J, *K18-hACE2* and *ACE2* mice were anaesthetized with intraperitoneal injection of 150 μl ketamine (10 mg ml^−1^)/xylazine (1 mg ml^−1^) solution. Hamsters were injected with 200 μl of ketamine (75 mg ml^−1^)/xylazine (5 mg ml^−1^ in PBS) solution. In total, 10^3^ or 10^5^ PFU of SARS-CoV-2 were administered intranasally in a total volume of 50 μl PBS per mouse, 100 μl PBS per hamster, delivered to both nostrils equally. All infection experiments were performed in the NYU BSL3 facility.

### SARS-CoV-2-infected lung and trachea RNA extraction and quantification

One lobe of lung was immersed in 1 ml Trizol solution (Invitrogen, 15596018) in Lysing Matrix A homogenization tubes (MP Biomedicals) immediately after dissecting from euthanized mouse or hamster. Lung was homogenized following manufacturer’s instructions (MP Biomedicals, FastPrep-24 5 G). Trachea was dissected and immersed in 1 ml PBS in a 2-ml microcentrifuge tube (Fisherbrand, 14-666-315) containing 1 stainless steel bead (QIAGEN, 69989). After the homogenization, PBS homogenates were centrifuged for 2 min at 5,000*g*. Five-hundred microlitres of homogenates were transferred and mixed with 500 μl Trizol solution for RNA extraction. Processing lung and trachea samples by the following steps: 200 μl of chloroform per 1 ml of Trizol reagent was added and vortexed thoroughly. Tubes were centrifuged at 12,000*g* for 10 min at 4 °C. Aqueous phase was transferred to a new RNase-free 1.5-ml tube. Total RNA was precipitated by adding 500 μl of isopropanol per 1 ml Trizol solution, and pelleted by centrifugation at 12,000*g* for 10 min at 4 °C. RNA pellet was washed with 500 μl of 75% ethanol once, air-dried at room temperature for 10 min, and dissolved with 100 μl of RNase-free water. Total RNA from SARS-CoV-2-infected lung or trachea was subjected to one-step real-time reverse transcription PCR using One-step PrimeScript RT–PCR kit (Takara, RR064B). Multiplex PCR was performed to detect SARS-CoV-2 nucleocapsid gene and mouse *Actb* gene. Probe targeting SARS-CoV-2 was labelled with FAM fluorophore and probes targeting *Actb* gene was labelled with Cy5 fluorophore (Supplementary Table 5). RT–PCR was performed on a LightCycler 480 instrument. SARS-CoV-2 RNA level was normalized to *Actb*.

### Lung RNA sequencing and analysis

Lung total RNA quality and quantity were examined using a Bioanalyzer (Agilent 2100, RNA 6000 nano kit). Sequencing libraries were constructed using a TruSeq Stranded Total RNA Library Prep Gold kit (Illumina, 20020599). Libraries were sequenced on an Illumina NovaSeq 6000 using a SP100 reagent kit (v1.5, 100 cycles). RNA-sequencing data were analysed by using the sns rna-star pipeline. In brief, adapters and low-quality bases were trimmed using Trimmomatic (v0.36). Sequencing reads were mapped to the mouse reference genome (mm10) using the STAR aligner (v2.7.3). Alignments were guided by a Gene Transfer Format (GTF) file. The mean read insert sizes and their standard deviations were calculated using Picard tools (v.2.18.20). The genes–samples counts matrix was generated using featureCounts (v1.6.3), normalized based on their library size factors using DEseq2, and differential expression analysis was performed. The read per million (RPM)-normalized BigWig files were generated using deepTools (v.3.1.0). Data were visualized using GraphPad Prism 9 or Rstudio.

### Plaque assay

The second lobe of lung or trachea was immersed in 1 ml PBS in a 2-ml microcentrifuge tube (Fisherbrand, 14-666-315) containing 1 stainless steel bead (5 mm, QIAGEN, 1026563) immediately after dissecting the SARS-CoV-2-infected mouse or hamster. Lung or trachea was homogenized following manufacturer’s instructions (TissueLyser II, QIAGEN, 85300). Homogenates were then centrifuged for 2 min at 5,000*g* and immediately frozen until plaque assay was performed. Plaque assay was performed with VeroE6 cells (ATCC, CRL-1586) plated in 24-well plates. Samples were diluted logarithmically in Minimal Essential Media (Gibco, 11095072), of which 200 μl were inoculated per well and incubated for 1 h at 37 °C. Inoculated cells were then overlayed with DMEM supplemented with 4% FBS, 1% penicillin-streptomycin-neomycin, and 0.2% agarose (Lonza, 50100). Overlayed cells were incubated at 37 °C for 48 h and subsequently fixed with 10% neutral buffered formalin for 24 h. Remaining VeroE6 cells were stained with 0.2% crystal violet in 20% ethanol for 10 min.

### Histology

The accessary lung lobes were immersed in 5 ml of 10% formalin solution (Sigma-Aldrich, HT501128) for 24 h at room temperature, and processed through graded ethanol, xylene and paraffin in a Leica Peloris automated processor. Five-micron paraffin-embedded sections were either stained with haematoxylin (Leica, 3801575) and eosin (Leica, 3801619) on a Leica ST5020 automated histochemical stainer or immunostained on a Leica BondRX autostainer, according to the manufacturers’ instructions. In brief, sections for immunostaining underwent epitope retrieval for 20 min at 100 °C with Leica Biosystems ER2 solution (pH 9.0, AR9640). Sections were incubated with one of the two ACE2 antibodies (Thermo, MA5-32307, clone SN0754 or Abcam, ab108209, clone EPR4436) diluted 1:100 for 30 min at room temperature and detected with the anti-rabbit HRP-conjugated polymer and DAB in the Leica BOND Polymer Refine Detection System (DS9800). Alternatively, sections were blocked with Rodent Block (Biocare, RBM961 L) prior to a 60-min incubation with SARS-CoV-2 nucleocapsid protein antibody (Thermo, MA1-7404, clone B46F) diluted 1:100 and then a 10-min incubation with a mouse-on-mouse HRP-conjugated polymer (Biocare MM620 H) and DAB (3,3′-diaminobenzidine). Sections were counter-stained with haematoxylin and scanned on either a Leica AT2 or Hamamatsu Nanozoomer HT whole slide scanner.

### ELISA

Mouse blood was collected via cardiac puncture, and isolated serum was diluted 100-fold using the dilution buffer of a mouse anti-SARS-CoV-2 antibody IgG titre serologic assay kit (ACROBiosystems, RAS-T023). Diluted samples were added to a microplate with pre-coated SARS-CoV-2 spike protein (2 μg ml^−1^), and incubated at 37 °C for 1 h. Following 3 washes, 100 μl of HRP-goat anti-mouse IgG (80 ng ml^−1^) was added to the microplate and incubated at 37 °C for 1 h. Following another 3 washes, 100 μl of substrate solution was added and incubated 37 °C for 20 min. The reaction was stopped by adding 50 μl stop solution, the absorbance was measured at 450 nm and 630 nm using an imaging reader (BioTek, Cytation 5 instrument, GEN5 software). Absorbance values for the serum samples were calculated by subtracting *A*_630 nm_ from *A*_450 nm_. A standard curve was generated using a series of diluted anti-SARS-CoV-2 mouse IgG control samples. Anti-SARS-CoV-2 mouse IgG titre in mouse serum was quantified using a standard curve.

### Statistics and reproducibility

RT–qPCR data are shown as mean ± s.d. of three technical replicates. SARS-CoV-2 levels in the infected mice are shown as mean ± s.e.m. GraphPad Prism 9 was used for statistical data analysis. Box plots contain 25th to 75th percentiles of the data, the horizontal line in each box denotes the median value, whiskers represent minima (low) and maxima (high). mSwAP-In engineering was repeated at least twice at each genomic locus described in this study.

### Biological materials availability statement

The Biological materials generated during and/or analysed during the current study are available from the corresponding author on reasonable request.

### Research animals statement

All experimental procedures were approved by the Institutional Animal Care and Use Committee (IACUC) at NYU Langone Health.

### Reporting summary

Further information on research design is available in the Nature Portfolio Reporting Summary linked to this article.

## Online content

Any methods, additional references, Nature Portfolio reporting summaries, source data, extended data, supplementary information, acknowledgements, peer review information; details of author contributions and competing interests; and statements of data and code availability are available at 10.1038/s41586-023-06675-4.

### Supplementary information

Supplementary Information This file contains Supplementary Figs. 1–3 and Supplementary Tables 1–5.

Reporting Summary

Supplementary File 1 Bamintersect analysis of capture sequenced clones.

Supplementary File 2 DEGs list for wild-type, *K18-hACE2* and 116 kb-*ACE2* lungs.

Supplementary File 3 Primer sequences for payload assemblies in a –*separate file*.

## Data Availability

Sequencing data including DNA sequencing, RNA sequencing, ChIP–seq and ATAC–seq are available in the Gene Expression Omnibus (GEO) database under accession number GSE235164. DNase-seq data were obtained from ENCODE https://www.encodeproject.org for small intestine (DS20770). Human reference genome hg38 and mouse reference genome mm10 are from UCSC genome browser https://genome.ucsc.edu. Resources generated in this study were or are being deposited to public repositories (Addgene accession numbers 208113–208117 and The Jackson Laboratories).
