# Supplementary material for: Mouse genome rewriting and tailoring of three important disease loci
Source: Nature. 2023 Nov 1;623(7986):423–31. doi: 10.1038/s41586-023-06675-4 (PMC10632133; doi:10.1038/s41586-023-06675-4)
Supplement: Supplementary file 1 — This file contains Supplementary Figs. 1–3 and Supplementary Tables 1–5. [file 41586_2023_6675_MOESM1_ESM.pdf]

nature portfolio

https://doi.org/10.1038/s41586-023-06675-4

Supplementary information

Mouse genome rewriting and tailoring of three important disease loci

In the format provided by the authors and unedited

Supplementary Information

Mouse genome rewriting and tailoring of three important disease loci

Weimin Zhang, Ilona Golynker, Ran Brosh, Alvaro Fajardo, Yinan Zhu, Aleksandra M. Wudzinska, Raquel Ordoñez, André M. Ribeiro-dos-Santos, Lucia Carrau, Payal Damani-Yokota, Stephen T. Yeung, Camille Khairallah, Antonio Vela Gartner, Noor Chalhoub, Emily Huang, Hannah J. Ashe, Kamal M. Khanna, Matthew T. Maurano, Sang Yong Kim, Benjamin R. tenOever, Jef D. Boeke

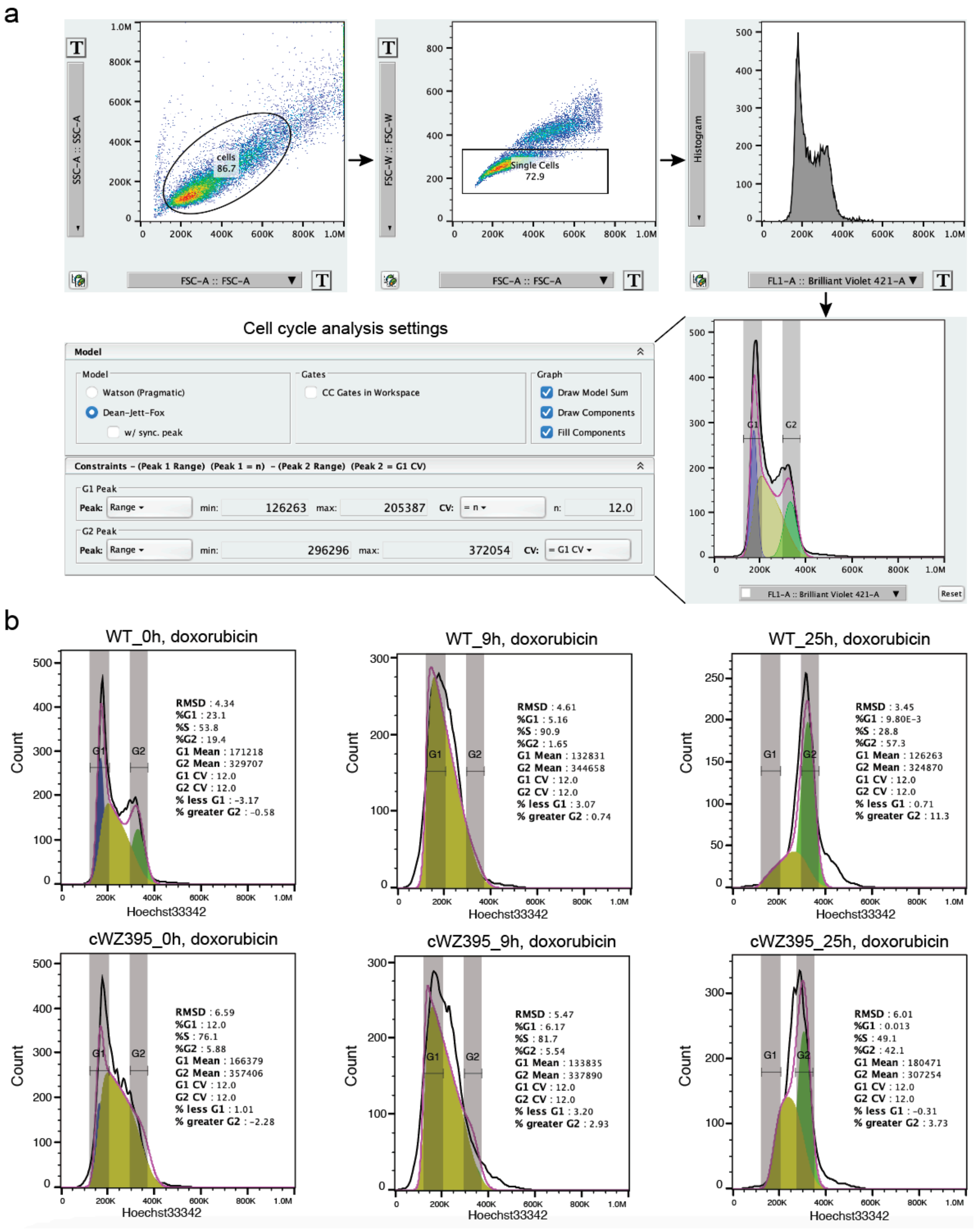

Supplementary Figure 1. (a) Wild type and *synTrp53* mESCs cell cycle arrest flow cytometry gating strategy. **(b)**. Cell cycle analysis using FlowJo's build-in tool, wild-type *Trp53* (MK6) without doxorubicin treatment was used for defining the G1 and G2 gates.

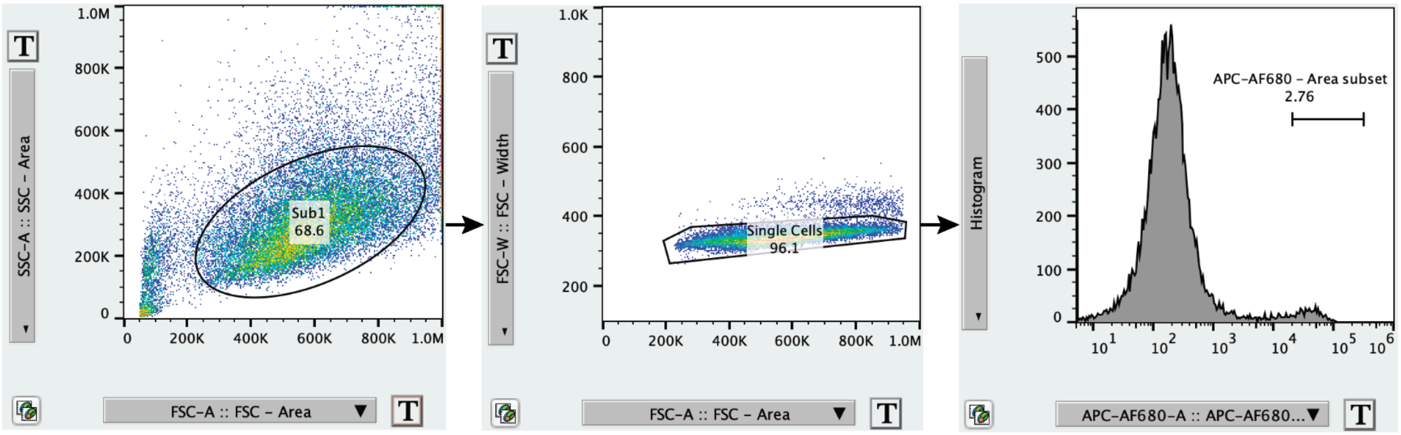

Supplementary Figure 2. Wild type and *synTrp53* mESCs cell apoptosis analysis flow cytometry gating strategy.

Fig. 4c. *hACE2* testis, panel 1

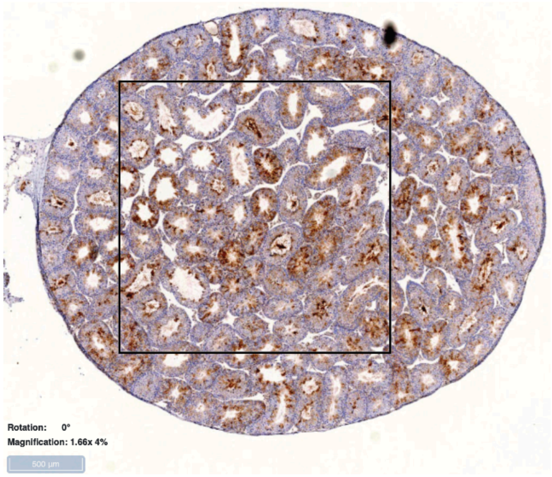

Fig. 4c. WT testis, panel 2

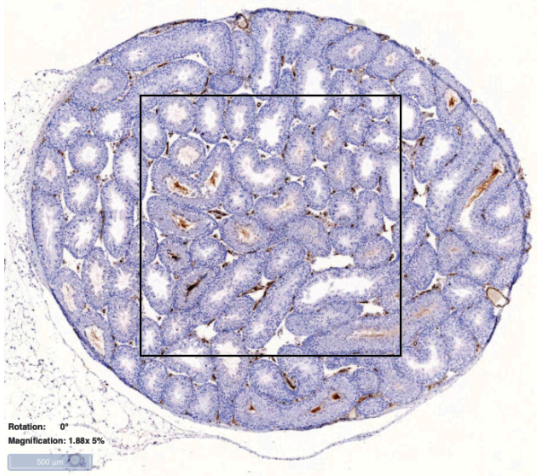

Fig. 4c. *hACE2* lung, panel 3

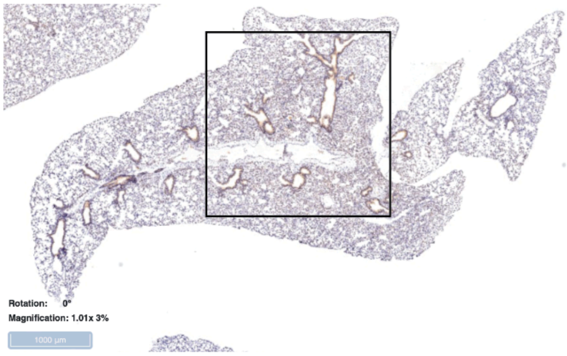

Fig. 4c. WT lung, panel 4

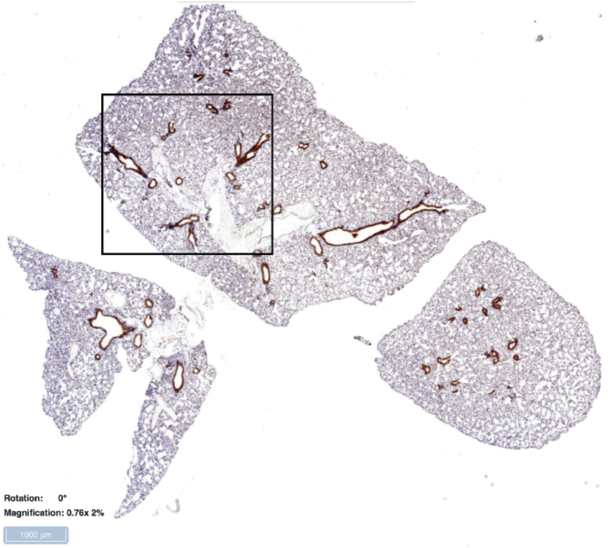

Fig. 5f. WT lung, panel 1

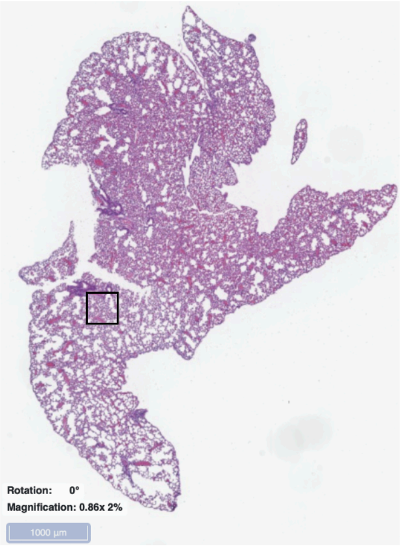

Fig. 5f. *K18-hACE2* lung, panel 2

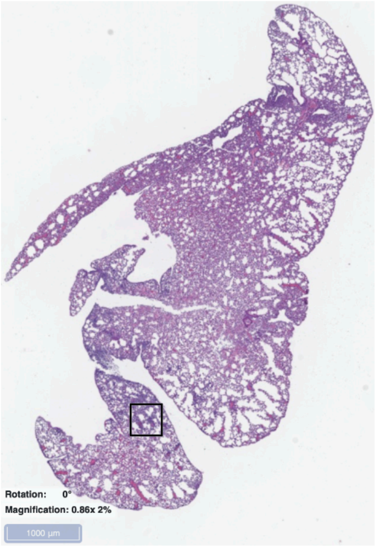

Fig. 5f. *hACE2* lung, panel 3

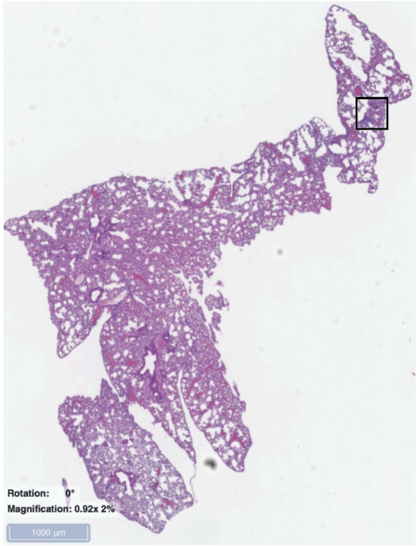

Supplementary Figure 3. Uncropped lungs, testes immunohistochemistry staining with ACE2 antibody, and H&E staining of infected lungs.

Supplementary Table 1. PCRTag and insertion coordinates for the subsequent mSwAP-In of *synTrp53*.

Name	Coordinates	Inserted sequences
PCRTag-1	Chr11: 69,597,884	GCTTTGAGCTGACAATTTGCCACCACCG
PCRTag-2	Chr11: 69,611,965	TCTGACGCCGTGGCTACTAACGGCAATA
PCRTag-3	Chr11: 69,625,010	TCTCTCCTGCCAGATGACACCTGAAGTG
PCRTag-4	Chr11: 69,637,459	CAAGGTTGACTTAAAAGCAGCTCTAGCG
PCRTag-5	Chr11: 69,650,231	CCCAAGCGTCATCATTAGCGGCAGTAAA
PCRTag-6	Chr11: 69,663,762	GTAAGCGCTTCTACGGGTGATACTTCTA
PCRTag-7	Chr11: 69,676,478	GGTGCCACTTGATAAGCTAGTTTGATCG
PCRTag-8	Chr11: 69,689,789	CGAAACCTTGAGTGGCTTGAGAATCGTC

Supplementary Table 2. Summary of tetraploid complementation injection success rate.

Tetraploid injected mESC clones	No. of Injected Embryos	No. of Pups	Birth Rate
116 kb *hACE2*-cWZ271	25	5	20%
116 kb *hACE2*-cWZ272	25	2	8%
180 kb *hACE2*-cWZ329	25	5	20%
180 kb *hACE2*-cWZ328	20	6	30%
180 kb *hACE2*-cWZ323	25	5	20%
TMPRSS2+116 kb *hACE2*-cWZ529	23	3	13%

Supplementary Table 3. Guide RNA sequences.

Guide RNA purposes	Sequences
gRNA targets UGT1	GCUUCAUGUGGUCGGGGUAG
gRNA targets UGT2	CACGAGGGUGGGCCAGGGCA
gRNA mediates MC1 insertion for *ACE2* humanization	AGGGUCUUCUCUACUCAAGG
Custom gRNA for *ACE2* humanization	UUAUUACUAGAGUAGCAGGG
gRNA mediates MC1 insertion for "CG-less" *Trp53* engineering	UACUGCCGUGUAUCGUAUUG
Custom gRNA for *synTrp53* engineering	UUGUAUAGGACCCUCGGGCA
Custom gRNA for engineering the 40 kb payload downstream of *synTrp53*	CAUCUCACCAGCCUAGCAGG
Custom gRNA for engineering the 75 kb payload downstream of *synTrp53*	UCAUUAACCCAGGAGCCACG
Custom gRNA for engineering the 115 kb payload downstream of *synTrp53*	ACCUGCUUCACAGAUAACUG

Supplementary Table 4. Amplicon sequencing primers.

Name	Sequence	Notes
oWZ1995	CTGGAGTTCAGACGTGTGCTCTTCCGATCTNNNNNNNNNNCACCATCGGAGCAGCGCTCATGG	R155R172 P7 primer with UMI
oWZ1996	CTGGAGTTCAGACGTGTGCTCTTCCGATCTNNNNNNNNNNGCGGAAATTTTCTTCTTCTGTACG	R270R279 P7 primer with UMI
oWZ1997	TTTCCCTACACGACGCTCTTCCGATCTNCACCTCCAGCTGGGAGCCGTGTC	R155R172 P5 primer for *Trp53*$^{wt/wt}$
oWZ1998	TTTCCCTACACGACGCTCTTCCGATCTNcTCCTGCATGGGGGGCATGAAC	R245R246 P5 primer for *Trp53*$^{wt/wt}$
oWZ2002	TTTCCCTACACGACGCTCTTCCGATCTNGCTTTCTTACTGCCTTGTGCTGGT	R270R279 P5 primer for *Trp53*$^{wt/wt}$
oWZ2004	CTGGAGTTCAGACGTGTGCTCTTCCGATCTNNNNNNNNNNCGCGCCTTCCTACCTGGAGT	R245R246 P7 primer with UMI
oWZ2006	TTTCCCTACACGACGCTCTTCCGATCTNNCACCTCCAGCTGGGAGCCGTGTC	R155R172 P5 primer for *Trp53*$^{syn/syn}$
oWZ2007	TTTCCCTACACGACGCTCTTCCGATCTNNcTCCTGCATGGGGGGCATGAAC	R245R246 P5 primer *Trp53*$^{syn/syn}$
oWZ2008	TTTCCCTACACGACGCTCTTCCGATCTNNGCTTTCTTACTGCCTTGTGCTGGT	R270R279 P5 primer *Trp53*$^{syn/syn}$

Supplementary Table 5. RT-qPCR primers and probes.

Name	Sequence	Notes
oWZ1588	GGACCCAGGAAATGTTCAGA	Human *ACE2* qPCR primer F1
oWZ1589	GGCTGCAGAAAGTGACATGA	Human *ACE2* qPCR primer R1
oWZ1590	GGGATCAGAGATCGGAAGAAGAAA	Human *ACE2* qPCR primer F2
oWZ1591	AGGAGGTCTGAACATCATCAGTG	Human *ACE2* qPCR primer R2
oWZ1592	AAACATACTGTGACCCCGCAT	Human *ACE2* qPCR primer F3
oWZ1593	CCAAGCCTCAGCATATTGAACA	Human *ACE2* qPCR primer R3
oWZ1596	TCCATTGGTCTTCTGCCATCC	Mouse *ACE2* qPCR primer F1
oWZ1597	AACGATCTCCCGCTTCATCTC	Mouse *ACE2* qPCR primer R1
oWZ1598	TGATGAATCAGGGCTGGGATG	Mouse *ACE2* qPCR primer F2
oWZ1599	ATTCTGAAGTCTCCGTGTCCC	Mouse *ACE2* qPCR primer R2
oWZ1600	GATCAAGATCATTGCTCCTCCTGA	Mouse *Actb* reference primer for RT-qPCR
oWZ1601	AAGGGTGTAAAACGCAGCTCA	Mouse *Actb* reference primer for RT-qPCR

oWZ1655	AGTCCGCCTAGAAGCACTTGCGGTG	*Actb* probe labeled with Cy5
N1-F	GACCCCAAAATCAGCGAAAT	SARS-CoV-2 nucleocapsid forward primer
N1-R	TCTGGTTACTGCCAGTTGAATCTG	SARS-CoV-2 nucleocapsid reverse primer
N1-probe	ACCCCGCATTACGTTTGGTGGACC	SARS-CoV-2 nucleocapsid probe labeled with FAM
oWZ1683	CCTCATTAGCTTTGTCACACGAGCC	Mouse *Actb* reference primer for copy number qPCR
oWZ1684	CCATAGGACTCCCTTCTATGAGCTG	Mouse *Actb* reference primer for copy number qPCR
oWZ1771	ATCGGGTGACAGAAGACCAATGGA	*ACE2* short isoform detection primer
oWZ1772	GCTTGTGAGAGCCTTAGGTTGGATT	*ACE2* short isoform detection primer
oWZ1781	GTCAGCCAACTTTTTCCCAAGAGTG	*ACE2* long transcript variant detection primer
oWZ1782	CCGTATCAATGATGCTTTCCGTCTG	*ACE2* long transcript variant detection primer
oWZ1826	CATCCTGGTGAGGAACGAAAGG	*Isg15* mouse RT-qPCR primer-F
oWZ1827	CTCAGCCAGAACTGGTCTTCGT	*Isg15* mouse RT-qPCR primer-R
oWZ1828	CCGAGTAACGGCTGCGACAAAG	*Cxcl11* mouse RT-qPCR primer-F
oWZ1829	CCTGCATTATGAGGCGAGCTTG	*Cxcl11* mouse RT-qPCR primer-R
oWZ1830	TGGACATTGCTACCACAGAGGC	*Mx1* mouse RT-qPCR primer-F
oWZ1831	TTGCCTTCAGCACCTCTGTCCA	*Mx1* mouse RT-qPCR primer-R
oWZ1855	CCTCTAACTGGTGTGATGGCGT	Human *TMPRSS2* RT-qPCR primer-F
oWZ1856	TGCCAGGACTTCCTCTGAGATG	Human *TMPRSS2* RT-qPCR primer-R
oWZ1857	AAGTCCTCAGGAGCACTGTGCA	Mouse *Tmprss2* RT-qPCR primer-F
oWZ1858	CAGAACCTCCAAAGCAAGACAGC	Mouse *Tmprss2* RT-qPCR primer-R
oWZ2100	GTGAAAGCGGGTGTGAGGAgc	Human *TMPRSS2* isoform 1 RT-qPCR primer forward
oWZ2101	CTGTGCGGGATAGGGGTTTTcc	Human *TMPRSS2* isoform 1 RT-qPCR primer reverse
oWZ2102	GCGAGGGGCGGGGAGCGCCg	Human *TMPRSS2* isoform 2 RT-qPCR primer forward
oWZ2103	cgGGTAGTACTGAGCCGGATGC	Human *TMPRSS2* isoform 2 RT-qPCR primer reverse
