# Supplementary material for: Mouse genome rewriting and tailoring of three important disease loci
Source: Nature. 2023 Nov 1;623(7986):423–31. doi: 10.1038/s41586-023-06675-4 (PMC10632133; doi:10.1038/s41586-023-06675-4)
Supplement: Supplementary file 2 — Reporting Summary [file 41586_2023_6675_MOESM2_ESM.pdf]

nature portfolio

Corresponding author(s): Jef D. Boeke

Last updated by author(s): Sep 4, 2023

Reporting Summary

Nature Portfolio wishes to improve the reproducibility of the work that we publish. This form provides structure for consistency and transparency in reporting. For further information on Nature Portfolio policies, see our Editorial Policies and the Editorial Policy Checklist.

Statistics

For all statistical analyses, confirm that the following items are present in the figure legend, table legend, main text, or Methods section.

n/a	Confirmed	
☐	☒	The exact sample size (*n*) for each experimental group/condition, given as a discrete number and unit of measurement
☐	☒	A statement on whether measurements were taken from distinct samples or whether the same sample was measured repeatedly
☐	☒	The statistical test(s) used AND whether they are one- or two-sided<br>*Only common tests should be described solely by name; describe more complex techniques in the Methods section.*
☒	☐	A description of all covariates tested
☐	☒	A description of any assumptions or corrections, such as tests of normality and adjustment for multiple comparisons
☐	☒	A full description of the statistical parameters including central tendency (e.g. means) or other basic estimates (e.g. regression coefficient) AND variation (e.g. standard deviation) or associated estimates of uncertainty (e.g. confidence intervals)
☐	☒	For null hypothesis testing, the test statistic (e.g. *F*, *t*, *r*) with confidence intervals, effect sizes, degrees of freedom and *P* value noted<br>*Give P values as exact values whenever suitable.*
☒	☐	For Bayesian analysis, information on the choice of priors and Markov chain Monte Carlo settings
☒	☐	For hierarchical and complex designs, identification of the appropriate level for tests and full reporting of outcomes
☒	☐	Estimates of effect sizes (e.g. Cohen's *d*, Pearson's *r*), indicating how they were calculated

Our web collection on statistics for biologists contains articles on many of the points above.

Software and code

Policy information about availability of computer code

Data collection	LightCycler 480 (Roche) software was used for RT-qPCR data collection. Gen5 (BioTek) software was used for absorbance data collection. Evos 5000 imaging system was used for collecting images. SONY Cell Analyzer or SH800s software were used for flow cytometry data collection. CRISPOR (Version 4.98, Version 4.99, Version 5.00) open source tool was used for all the guide RNA information collection.
Data analysis	RNA sequencing data were analyzed using sns rna-star pipline (https://igordot.github.io/sns/routes/rna-star.html), which include the following tools: Trimmomatic (v0.36), STAR aligner (v2.7.3), featureCounts (v1.6.3) and deepTools (v.3.1.0) were used for . bcl2fastq (v2.20), Trimmomatic (v0.39), BWA (v0.7.17), samblaster (v0.1.24) and BEDOPS (v2.4.35) were used for DNA sequencing data analysis. fastp tool was used for filtering low quality amplicon seq reads. UMI-tools v1.0.1 was used for UMI sequence extraction, GraphPad Prism 9 was used for statistical data analysis. Omero Plus was used for histology data analysis. FlowJo was used for flow cytometry data analysis.

For manuscripts utilizing custom algorithms or software that are central to the research but not yet described in published literature, software must be made available to editors and reviewers. We strongly encourage code deposition in a community repository (e.g. GitHub). See the Nature Portfolio guidelines for submitting code & software for further information.

Data

Policy information about availability of data

All manuscripts must include a data availability statement. This statement should provide the following information, where applicable:

- Accession codes, unique identifiers, or web links for publicly available datasets
- A description of any restrictions on data availability
- For clinical datasets or third party data, please ensure that the statement adheres to our policy

Sequencing data including DNA-seq, RNA-seq, ChIP-seq and ATAC-seq are available in the Gene Expression Omnibus (GEO) database, accession number GSE235164. DNase-seq data were obtained from ENCODE https://www.encodeproject.org for small intestine (DS20770). Human reference genome hg38 and mouse reference genome mm10 are from UCSC genome browser https://genome.ucsc.edu. All data are available in the main text or extended data. Resources generated in this study are deposited to public repositories (Addgene, The Jackson Labs).

Research involving human participants, their data, or biological material

Policy information about studies with human participants or human data. See also policy information about sex, gender (identity/presentation), and sexual orientation and race, ethnicity and racism.

Reporting on sex and gender	N/A
Reporting on race, ethnicity, or other socially relevant groupings	N/A
Population characteristics	N/A
Recruitment	N/A
Ethics oversight	N/A

Note that full information on the approval of the study protocol must also be provided in the manuscript.

Field-specific reporting

Please select the one below that is the best fit for your research. If you are not sure, read the appropriate sections before making your selection.

☒ Life sciences ☐ Behavioural & social sciences ☐ Ecological, evolutionary & environmental sciences

For a reference copy of the document with all sections, see nature.com/documents/nr-reporting-summary-flat.pdf

Life sciences study design

All studies must disclose on these points even when the disclosure is negative.

Sample size	Sample size was not predetermined for the in vitro cell culture experiments, including synTrp53 and its subsequent genome writing, ACE2 humanization in mESCs. We reported the number of screened colonies and the number of winners for each experiment. For RT-qPCR of SARS-CoV-2 infected animals, sample size was determined by animal sample size, animal sample sizes were chosen to enable significant statistical power while minimizing unnecessary wastage. Three RT-qPCR technical replicates were performed based on the standard in the field.<br>For the first qualitative SARS-CoV-2 infection experiment, with the goal of identifying an optimal infection titer (10^3PFU or 10^5PFU), we minimized the animal number by using 1 male and 1 female for each genotype (2 female K18-ACE2 mice were used for the 10^3PFU group,because the male K18-ACE2 mice were wounded due to fights). For the subsequent hACE2 mouse and golden hamster infections, 4 or 5 animals were used for each group to achieve statistical significance. For the longitudinal SARS-CoV-2 infection experiment, 9 animals for each genotype were infected and 3 were sacrificed for tissue collections at each post-infection time point. For human TMPRSS2 isoform 1 and 2 detection, one animal was used for 9 tissues collection, RT-qPCR was performed with 3 technical replicates. For CUT&RUN and ATAC-seq of testicular or small intestinal cells, two animals were used as biological replicates for each genotype. When analyzing the dACE2 isoform levels in the infected and uninfected lungs, 3 uninfected mice were used as control, all the infected 116kb-hACE2 (8) were used an unpaired two-tailed, Mann-Whitney t test was used. For biallelic TMPRSS2 humanization, three different founder mESC lines were used to rule out founder effect.
Data exclusions	When calculating the mSwAP-In efficiency for synTrp53,two mESC clones were excluded due to multiple copies of synTrp53 payload were detected in the subsequent capture-sequencing analysis.
Replication	The mSwAP-In engineering of Trp53, ACE2 and TMPRSS2 were performed at least three times with similar success rate each time. SARS-CoV-2 infection of hACE2 mice were performed four times, each time with K18-ACE2 mice as positive infection control as well as wild-type mice as negative infection control.
Randomization	Similar aged mice and hamsters were selected randomly for the infection experiments.

Blinding	Investigators were not blinded to infected animal groups due to data collection and analysis in the BSL3 facility.

Reporting for specific materials, systems and methods

We require information from authors about some types of materials, experimental systems and methods used in many studies. Here, indicate whether each material, system or method listed is relevant to your study. If you are not sure if a list item applies to your research, read the appropriate section before selecting a response.

Materials & experimental systems

n/a	Involved in the study
☐	☒ Antibodies
☐	☒ Eukaryotic cell lines
☒	☐ Palaeontology and archaeology
☐	☒ Animals and other organisms
☒	☐ Clinical data
☒	☐ Dual use research of concern
☒	☐ Plants

Methods

n/a	Involved in the study
☐	☒ ChIP-seq
☐	☒ Flow cytometry
☒	☐ MRI-based neuroimaging

Antibodies

Antibodies used	ACE2 antibodies: Thermo, MA5-32307 clone SN0754; Abcam, ab108209 clone EPR4436. SARS-CoV-2 Nucleocapsid antibody: Thermo, MA1-70404 clone B46F. Histone H3K4me3 antibody: EpiCypher 13-0041. Histone H3K27ac antibody: EpiCypher 13-0045. Mouse anti-SARS-CoV-2 IgG (Anti-Spike trimer antibody): ACROBiosystems, RAS-T023.
Validation	All the primary antibodies are validated by manufactures' quality control process. ACE2 (rabbit monoclonal) antibody MA5-32307 (species reactivity: human, mouse) was validated in the following publications, PMID: 33147445, PMID: 34274504, PMID: 33568991. ACE2 (rabbit monoclonal) antibody ab108209 (species reactivity: human) was validated in the following publications, PMID: 32485164, PMID: 33113348, PMID: 34214142. SARS-CoV-2 Nucleocapsid (mouse monoclonal) antibody (MA1-7404) was validated in the following publications, PMID: 33232663, PMID: 33200131, PMID: 32647285. H3K27ac antibody was validated in the following publications, PMID: 26004229, PMID: 30244833. H3K4me3 antibody was validated in the following publications, PMID: 35210568, PMID: 30244833. Mouse anti-SARS-CoV-2 IgG (Anti-Spike trimer antibody) is provided in the ELISA kit (ACROBiosystems, RAS-T023), which was validated in the following publication, PMID: 35486845.

Eukaryotic cell lines

Policy information about cell lines and Sex and Gender in Research

Cell line source(s)	The C57BL/6J male mESC line (MK6) was obtained from NYU Langone Health Rodent Genetic Engineering Lab.<br>Mouse embryonic fibroblast (MEF) cells were purchased from CellBiolabs (CBA-310), and were subsequently inactivated via mitomycin-C treatment.<br>VeroE6 cells were purchased from ATCC (CRL-1586).
Authentication	The karyotype of C57BL/6J male mESC line (MK6) was validated using ddPCR assay (Primers and probes are from Codner et al. PMID: 27496052). The pluripotency of C57BL/6J male mESC line was validated for tetraploid complementation competence in NYU Langone Health Rodent Genetic Engineering Lab. Mouse embryonic fibroblast (MEF) cells were validated by manufacture's quality control process, mainly about maintaining the pluripotent status of mESCs. VeroE6 cells were validated in multiple previous publications.
Mycoplasma contamination	All cell lines were negative for Mycoplasma test.
Commonly misidentified lines<br>(See ICLAC register)	No commonly misidentified cell line was used in this study.

Animals and other research organisms

Policy information about studies involving animals; ARRIVE guidelines recommended for reporting animal research, and Sex and Gender in Research

Laboratory animals	C57BL/6J-albino female mice (Charles River laboratories, strain#493) were used for harvesting blastocysts for chimeric mESC infection. B6D2F1/J (Jackson laboratories, strain#100006) mice were used for harvesting blastocysts for fusion to tetraploid blastocysts. Wild-type C57BL/6J (strain#000664) and K18-hACE2 (strain#034860) mice were obtained from The Jackson laboratory. Golden hamsters were obtained from Charles River Laboratories (strain#049). Mice and hamsters were housed in the NYU Langone Health BSL1 barrier facility. ~4 weeks old 116kb-hACE2 and wild-type mice were used for measuring hACE2 and mAce2 mRNA levels. ~10 weeks old hACE2 or wild-type mice were used for immunohistochemistry staining of lung and testis. ~36 weeks old male mice were used for testicular cells collection for the CUT&RUN assay. ~25 weeks old mice were used for small intestine cells collection for the ATAC-seq assay. 10-15 weeks old mice and 10-12 weeks old hamsters were transferred to the NYU Langone Health BSL3 facility

	for the SARS-CoV-2 infection. All animals were settled for at least two days in the NYU Langone Health BSL3 facility prior to the SARS-CoV-2 infection.
Wild animals	This study did not include wild animals.
Reporting on sex	Initially we infected both male and female mice, however, we did not notice a significant difference in terms of infectibility due to sex difference. Thus, we used female mice for the following experiments.
Field-collected samples	This study did not include samples collected from the field.
Ethics oversight	All experimental procedures were approved by the Institutional Animal Care and Use Committee (IACUC) at NYU Langone Health.

Note that full information on the approval of the study protocol must also be provided in the manuscript.

Plants

Seed stocks	N/A
Novel plant genotypes	N/A
Authentication	N/A

ChIP-seq

Data deposition

☒ Confirm that both raw and final processed data have been deposited in a public database such as GEO.

☒ Confirm that you have deposited or provided access to graph files (e.g. BED files) for the called peaks.

Data access links
May remain private before publication.

https://www.ncbi.nlm.nih.gov/geo/query/acc.cgi?acc=GSE235164

Files in database submission

Each sample has two processed files followed by two raw sequencing files:
WTtestis_1-IgG-BS19091A.mm10.density.bw
WTtestis_1-IgG-BS19091A.hg38_noalt.density.bw
BS19091A_R1_000.fastq.gz
BS19091A_R2_000.fastq.gz
WTtestis_2-IgG-BS19092A.mm10.density.bw
WTtestis_2-IgG-BS19092A.hg38_noalt.density.bw
BS19092A_R1_000.fastq.gz
BS19092A_R2_000.fastq.gz
116kACE2testis_1-IgG-BS19093A.mm10.density.bw
116kACE2testis_1-IgG-BS19093A.hg38_noalt.density.bw
BS19093A_R1_000.fastq.gz
BS19093A_R2_000.fastq.gz
116kACE2testis_2-IgG-BS19094A.mm10.density.bw
116kACE2testis_2-IgG-BS19094A.hg38_noalt.density.bw
BS19094A_R1_000.fastq.gz
BS19094A_R2_000.fastq.gz
180kACE2testis_1-IgG-BS19095A.mm10.density.bw
180kACE2testis_1-IgG-BS19095A.hg38_noalt.density.bw
BS19095A_R1_000.fastq.gz
BS19095A_R2_000.fastq.gz
180kACE2testis_2-IgG-BS19096A.mm10.density.bw
180kACE2testis_2-IgG-BS19096A.hg38_noalt.density.bw
BS19096A_R1_000.fastq.gz
BS19096A_R2_000.fastq.gz
WTtestis_1-H3K4me3-BS19097A.mm10.density.bw
WTtestis_1-H3K4me3-BS19097A.hg38_noalt.density.bw
BS19097A_R1_000.fastq.gz
BS19097A_R2_000.fastq.gz
WTtestis_2-H3K4me3-BS19098A.mm10.density.bw
WTtestis_2-H3K4me3-BS19098A.hg38_noalt.density.bw
BS19098A_R1_000.fastq.gz
BS19098A_R2_000.fastq.gz
116kACE2testis_1-H3K4me3-BS19099A.mm10.density.bw
116kACE2testis_1-H3K4me3-BS19099A.hg38_noalt.density.bw
BS19099A_R1_000.fastq.gz
BS19099A_R2_000.fastq.gz
116kACE2testis_2-H3K4me3-BS19100A.mm10.density.bw
116kACE2testis_2-H3K4me3-BS19100A.hg38_noalt.density.bw
BS19100A_R1_000.fastq.gz

BS19100A_R2_000.fastq.gz
180kACE2testis_1-H3K4me3-BS19101A.mm10.density.bw
180kACE2testis_1-H3K4me3-BS19101A.hg38_noalt.density.bw
BS19101A_R1_000.fastq.gz
BS19101A_R2_000.fastq.gz
180kACE2testis_2-H3K4me3-BS19102A.mm10.density.bw
180kACE2testis_2-H3K4me3-BS19102A.hg38_noalt.density.bw
BS19102A_R1_000.fastq.gz
BS19102A_R2_000.fastq.gz
WTtestis_1-H3K27ac-BS19103A.mm10.density.bw
WTtestis_1-H3K27ac-BS19103A.hg38_noalt.density.bw
BS19103A_R1_000.fastq.gz
BS19103A_R2_000.fastq.gz
WTtestis_2-H3K27ac-BS19104A.mm10.density.bw
WTtestis_2-H3K27ac-BS19104A.hg38_noalt.density.bw
BS19104A_R1_000.fastq.gz
BS19104A_R2_000.fastq.gz
116kACE2testis_1-H3K27ac-BS19105A.mm10.density.bw
116kACE2testis_1-H3K27ac-BS19105A.hg38_noalt.density.bw
BS19105A_R1_000.fastq.gz
BS19105A_R2_000.fastq.gz
116kACE2testis_2-H3K27ac-BS19106A.mm10.density.bw
116kACE2testis_2-H3K27ac-BS19106A.hg38_noalt.density.bw
BS19106A_R1_000.fastq.gz
BS19106A_R2_000.fastq.gz
180kACE2testis_1-H3K27ac-BS19107A.mm10.density.bw
180kACE2testis_1-H3K27ac-BS19107A.hg38_noalt.density.bw
BS19107A_R1_000.fastq.gz
BS19107A_R2_000.fastq.gz
180kACE2testis_2-H3K27ac-BS19108A.mm10.density.bw
180kACE2testis_2-H3K27ac-BS19108A.hg38_noalt.density.bw
BS19108A_R1_000.fastq.gz
BS19108A_R2_000.fastq.gz

Genome browser session
(e.g. UCSC)

bigWig graph files are included in the GEO submission at https://www.ncbi.nlm.nih.gov/geo/query/acc.cgi?acc=GSE235164

Methodology

Replicates

Two biological replicates were included from each mouse genotype.

Sequencing depth

The sequencing was paired-ends 36 bp, below lists the sequenced reads number (middle column), and the reads number that passed filtering (right column) for each sample:

Sample	Sequenced	Passed filtering
BS19091A	5,186,596	5,164,880
BS19092A	6,201,762	6,174,850
BS19093A	4,088,828	4,066,864
BS19094A	7,434,002	7,400,726
BS19095A	8,474,116	8,441,682
BS19096A	7,469,576	7,435,510
BS19097A	8,054,702	8,015,096
BS19098A	7,071,774	7,036,002
BS19099A	6,363,904	6,330,016
BS19100A	7,221,410	7,182,900
BS19101A	6,982,310	6,947,388
BS19102A	6,914,790	6,879,094
BS19103A	6,283,860	6,253,180
BS19104A	3,238,216	3,222,468
BS19105A	5,572,412	5,545,936
BS19106A	4,151,942	4,133,534
BS19107A	5,650,654	5,621,216
BS19108A	4,212,500	4,192,566

Antibodies

Histone H3K4me3 antibody: EpiCypher 13-0041. Histone H3K27ac antibody: EpiCypher 13-0045. IgG control antibody included in the EpiCypher CUT&RUN kit.

Peak calling parameters

We did not perform peak calling for the ChIP-seq dataset in this study, the bigWig files reflecting the coverage tracks were generated using BWA v0.7.17 aligner and BEDOPS.

Data quality

Two biological replicates were used for data quality insurance, and coverage tracks were compared with each other as shown in Fig. S7.

Software

ChIP seq data were visualized on the UCSC genome browser. The full processing pipeline is available at https://github.com/mauranolab/mapping

Flow Cytometry

Plots

Confirm that:

☒ The axis labels state the marker and fluorochrome used (e.g. CD4-FITC).

☒ The axis scales are clearly visible. Include numbers along axes only for bottom left plot of group (a 'group' is an analysis of identical markers).

☒ All plots are contour plots with outliers or pseudocolor plots.

☒ A numerical value for number of cells or percentage (with statistics) is provided.

Methodology

Sample preparation	Cells were treated with 250 nM doxorubicin (Tocris, 2252) for desired period. mESCs were trypsinized and APPROXIMATELY 10^6 cells were stained with 10ug/mL Hoechst33342 for 30 min at room temperature for DNA content-based cell cycle analysis, or stained with annexin V conjugated with 680 fluorophores for 15 min at room temperature for apoptosis analysis. Samples were kept on ice during the flow cytometry experiment.
Instrument	SONY SA3800, SONY SH800s
Software	SONY SA3800, SH800s, FlowJo 10.9.0
Cell population abundance	For hoechst33342 stained assay for cell cycle arrest analysis, near 100% of the cells were successfully stain when comparing to the no stain control. The cell population abundance in G1 S G2 phases were determined using FlowJo's build-in cell cycle analysis tool. When analyzing the apoptotic cell population, wild type or synTrp53 mESCs treated with doxorubicin were stained with Annexin V conjugated with 680 fluophor. The apoptotic cell population ranged from 2.76% to 21.9% of the population.
Gating strategy	FSC-area/SSC-area gate was drawn to include the most abundant regular sized and shaped cells. FSC-area/FSC-width gate was drawn to exclude the doublers. For the cell cycle arrest assay, unstained cells were used to establish the negative threshold. Hoechst positive population was displayed based on hoechst signal intensity to reflect cells in different cell cycle stages. For the cell apoptosis analysis, mESCs without doxorubicin treatment was used to establish negative gate, APC-680 was used to separate apoptotic cells and normal cells.

☒ Tick this box to confirm that a figure exemplifying the gating strategy is provided in the Supplementary Information.
